# Comparison of biological properties of human adipose tissue-derived mesenchymal stem/ stromal cells from healthy and diabetic donors: consequences for cell-based medicinal product development

**DOI:** 10.1186/s12933-025-02943-x

**Published:** 2025-10-29

**Authors:** Patrycja Dudek, Anna Łabędź-Masłowska, Zbigniew Madeja, Ewa Zuba-Surma

**Affiliations:** Department of Cell Biology, Faculty of Biochemistry, Biophysics and Biotechnology, Jagiellonian University, Kraków, Poland

**Keywords:** Adipose tissue, Mesenchymal stem/stromal cells, Cell-based medicinal products, Advanced therapy medicinal products, Diabetes, Donor qualification criteria, Tissue regeneration, Angiogenesis, Chondrogenesis

## Abstract

**Background:**

Diabetes mellitus is a civilisation disease that can cause damage to tissues and organs as well as affects the biological properties of cells isolated from these tissues. In recent years, there has been increasing interest in cell-based therapies, including the use of mesenchymal stem/stromal cells (MSCs). Therefore, the aim of the current study was to compare the biological potential of adipose tissue-derived MSCs (AT-MSCs) from healthy and diabetic donors under in vitro conditions and to clarify the implications for cell-based medicinal product development. Biological potential of both populations of AT-MSCs was also investigated in the relation to their major therapeutic mechanisms of action—we focused on the chondrogenic and osteogenic differentiation capacity of AT-MSCs and their pro-angiogenic potential.

**Methods:**

Human AT-MSCs derived from healthy and type 2 diabetes (T2D) donors underwent biological characterization including assessment of: morphology, viability, antigenic profile, proliferation, presence of senescent cells and oxidative stress, pro-angiogenic properties of AT-MSC secretome as well as trilineage differentiation potential in vitro. AT-MSCs were cultured under the control and diabetes mimicking culture conditions.

**Results:**

We observed no significant differences in morphology, viability, expression of MSC markers, proliferation rate, concentration of oxidative stress marker (8OHdG) and content of senescent cells between AT-MSCs from healthy and T2D donors under control culture conditions. The conditioned medium from a culture of diabetic AT-MSCs was found to improve the pro-angiogenic potential of human umbilical vein endothelial cells (HUVECs), compared with the medium from healthy AT-MSCs. HUVECs that were incubated in conditioned media collected from healthy AT-MSCs from diabetic culture conditions, exhibited greater potential to form capillary-like structures. Furthermore, diabetic culture conditions induced the oxidative stress in healthy AT-MSCs. Diabetic AT-MSCs exhibited greater chondrogenic differentiation capacity along with lower adipogenic differentiation potential and comparable osteogenic differentiation capacity when compared to healthy donor-derived AT-MSCs.

**Conclusions:**

The present study provides evidence of the biological potential of AT-MSCs from diabetic donors, which can be used as an active substance in the development of cell-based autologous advanced therapy medicinal products (ATMPs) dedicated for the treatment of e.g. osteoarthritis or myocardial infarction. Diabetic AT-MSCs in the used culture conditions are functional cells with greater chondrogenic and pro-angiogenic potential when compared to AT-MSCs from healthy donors. This increases the possibility of treating diabetic patients using their own cells.

**Graphical abstract:**

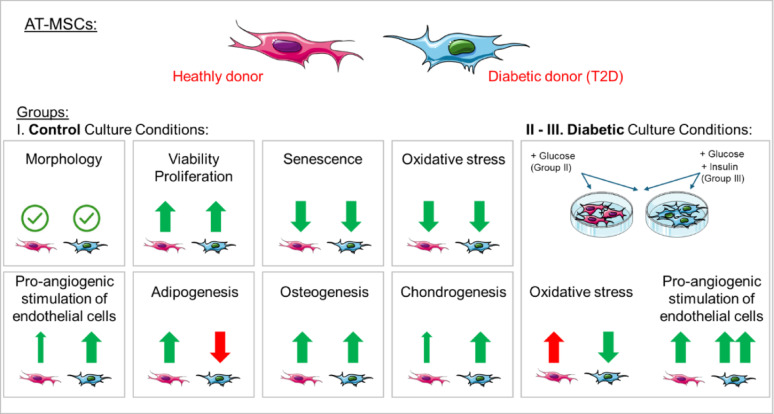

**Supplementary Information:**

The online version contains supplementary material available at 10.1186/s12933-025-02943-x.

## Research insights

**What is currently known about this topic?**

Diabetes in tissue donors can significantly affect the biological properties of cells isolated from their tissues. Before developing a cell-based medicinal product derived from diabetic tissue donors, it is essential to determine the functionality of the isolated cells, including their pro-regenerative mechanisms of action.

**What is the key research question?**

Are AT-MSCs from diabetic donors functional cells when compared to AT-MSCs from healthy donors?

**What is new?**

The present study provides evidence of the biological potential of AT-MSCs derived from diabetic donors, which can be used as an active substance in the development of cell-based medicinal products. Diabetic AT-MSCs are functional cells with greater chondrogenic and pro-angiogenic potential when compared to AT-MSCs from healthy donors.

**How might this study influence clinical practice?**

For patients with diabetes, it is possible to manufacture an autologous ATMP based on AT-MSCs, dedicated for treatment of *e.g.* osteoarthrosis or myocardial infarction.

## Background

Mesenchymal stem/stromal cells (MSCs) are one of the most extensively studied adult stem cell population due to their broad pro-regenerative activity observed in in vivo research and numerous clinical trials [1]. The pro-regenerative effects observed post MSC transplantation may result from their differentiation into tissue-specific cells (*e.g.* chondroblasts), paracrine signaling (*e.g.* pro-angiogenic, cytoprotective factors), the release of extracellular vesicles (EVs) or immunomodulation [2, 3]. MSCs can be isolated from bone marrow (BM), adipose tissue (AT), birth tissues, dental pulp or muscles [4, 5]. Because AT can contain 500 times more MSCs than BM [6], and its collection via liposuction is relatively non-invasive for patients compared to BM extraction, AT is the most suitable source of MSCs [7]. According to the International Society for Cellular Therapy (ISCT) position statement, the minimal characteristics of MSCs include: (i) adhesion to plastic surfaces in in vitro culture; (ii) expression of CD73, CD90 and CD105 and parallel the lack of expression of CD45, CD34, CD14 or CD11b, CD79α or CD19 and HLA-DR surface molecules; (iii) ability to differentiate into adipocytes, osteoblasts, or chondroblasts in vitro [8].

The Clinicaltrials.gov database currently has records for 538 completed clinical trials of MSC therapies (studies found for “mesenchymal stem cells”) intended for the treatment of several diseases including myocardial infarction and diabetes. In these clinical trials investigators used autologous or allogenic advanced therapy medicinal products (ATMPs) containing human MSCs as an active substance. However, prior to the development and use of ATMPs, a number of factors need to be considered, including biological potential of cells (active substance of ATMPs), the type of application: autologous vs. allogenic or tissue donor selection criteria, which can have a significant impact on patient outcome [9].

The effectiveness of ATMP therapy containing MSCs is influenced by the interactions of the cells with their microenvironment, which can significantly impact their function [9, 10]. These interactions may occur at two distinct stages: before the cells are isolated from the source tissue and after they are administered to the target tissue. It should also be noted that microenvironmental factors may be affected by the patient's condition, including diseases such as diabetes. Type 2 diabetes mellitus (T2D) is a civilizational disease that causes hyperglycemia which can alter the biological properties of cells isolated from various tissues [11, 12]. Importantly, some investigators reported abnormal characteristics or dysfunction of MSCs isolated from T2D tissue donors that can limit their therapeutic use [13, 14], while the others did not observe significant changes in their biological characteristics [15, 16]. Because the application of autologous MSCs minimizes the risk of immune rejection and the transfer of pathogen particles [9], it is essential to verify the potency of MSCs, especially derived from diabetic donors, taking into account *e.g.* their future autologous applications, in in vitro research before development of ATMPs or defining the tissue donor qualification criteria. Conducting this research will have a significant impact on the effectiveness of therapies using MSCs.

In the face of divergent scientific reports related with biological potential of diabetic MSCs, the main objective of the study was to evaluate the impact of T2D in tissue donor on the biological properties of AT-MSCs in comparison with AT-MSCs from healthy patients. In the study, we also focused on the chondrogenic and osteogenic differentiation capacity of AT-MSCs as well as their pro-angiogenic potential. The second objective was to investigate the biological properties of both types of AT-MSCs under in vitro conditions that mimic the microenvironment of diabetic tissue in patients who are treated with or without insulin (and which also correspond to conditions after cell administration), a topic that, to the best of our knowledge, has not been studied before.

The novelty and importance of the current study lay in demonstrating the effectiveness of diabetic MSCs, also in a diabetic microenvironment. This suggests that autologous cell applications can be used to treat diabetic patients, increasing the potential for treatment with their own cells. The current study examined the biological potential of diabetic AT-MSCs prior to the development of autologous ATMPs for diabetic patients and form the basis for including diabetic patients as adipose tissue donors for manufacturing autologous ATMPs.

## Methods

The extended description of the methods was included in Additional file 1.

### Culture of AT-MSCs

Human AT-MSCs (cryopreserved at primary passage) from healthy donors (n = 3, age range: 44–61; BMI mean: 29,7) and patients with T2D (n = 3, age range: 39–76; BMI mean: 30,7; Additional file 1, Table S1) were purchased from Lonza. According to the certificate of analysis provided by Lonza, AT-MSCs from each donor exhibited the following multiantigenic phenotype: CD13^+^/ CD29^+^/ CD44^+^/ CD73^+^/ CD90^+^/ CD105^+^/ CD166^+^/ CD14^−^/ CD31^−^/ CD34^−^/ CD45^−^. In the study, three types of culture medium (CM) were used: i) standard CM (Control)- αMEM medium supplemented with 5% human platelet lysate (hPL) MultiPL’100i (both from Macopharma), 2 IU/ml heparin (Polfa Warszawa S.A.), and 1% penicillin–streptomycin solution (P/S, Thermo Fisher Scientific), ii) standard CM with addition of 25 mM of glucose (Thermo Fisher Scientific) to mimic diabetic conditions, or iii) standard CM with addition of 25 mM glucose (Thermo Fisher Scientific) and 1 µg/ml insulin (Merck) to mimic insulin-treated diabetic conditions (Table 1). Cells were seeded in TC culture flasks (BD Falcon) in the listed three types of CM and further cultivated for 4–7 days prior to analysis including morphology, antigenic profile, viability, proliferation, oxidative stress, senescence, trilineage differentiation etc. according to the experimental scheme presented in the Additional file 1, Fig. S1. AT-MSCs were maintained under standard culture conditions (37 °C, 5% CO_2_, 95% humidity) in HERACELL VIOS 160i CO_2_ Incubator (Thermo Fisher Scientific). The AT-MSCs were passaged with Tryple Select Enzyme (Thermo Fisher Scientific) when the confluence of cells reached app. 80–90% and were used in the experiments from the fourth to the sixth passage. Each type of AT-MSC obtained from three individual human donors was used to replicate each experiment.

**Table 1:** Experimental groups, culture conditions and types of cells used in the study

Group	Cells	
	AT-MSCs derived from Healthy donors	AT-MSCs derived from T2D donors
Control	Culture medium (CM)*: αMEM medium (containing L-glutamine) supplemented with 5% hPL, 2 IU/ml heparin, 1% P/S	
	Differentiation medium (DM): StemPro: Adipogenesis Differentiation Kit, Chondrogenesis Differentiation Kit, Osteogenesis Differentiation Kit	
Glucose (presence of high glucose mimic diabetic tissue microenvironment)	CM/ DM* with addition of 25 mM glucose	
Glucose + Insulin (presence of high glucose + insulin mimic diabetic tissue microenvironment in donors/ recipients treated with insulin)	CM/ DM* with addition of 25 mM glucose and 1 µg/ml insulin	

### Antigenic phenotyping of AT-MSCs

The AT-MSCs were immunolabelled with antibodies against selected antigens: anti-CD19, anti-CD45, anti-CD90, anti-CD105 according to the manufacturer’s protocols for 30 min at 4 °C and analyzed using LSR Fortessa flow cytometer and FACS Diva software (Becton Dickinson).

### Apoptosis and necrosis detection

The evaluation of apoptosis and necrosis was conducted using the FITC Annexin V Apoptosis Detection Kit (BD Bioscience) according to the manufacturer’s protocol and analyzed using LSR Fortessa flow cytometer and FACS Diva software.

### Assessment of the proliferation rate

The AT-MSCs were seeded in a 96-well plate (Eppendorf) at the density of 10^3^ cells/well in the culture medium (Table 1). The analysis was carried out using the Cell Counting Kit-8 (CCK-8, Merck) according to the manufacturer’s protocol. The absorbance was measured at a wavelength of 450 nm using a Multiskan FC microplate photometer (Thermo Fisher Scientific).

### Proliferation efficiency

The AT-MSCs were seeded in a 6-well plates (Eppendorf) with a density of 10, 100, 200, 400 or 1000 cells per well in the culture medium (Table 1). After 21 days, cells were fixed with 4% paraformaldehyde (CHEMPUR) and stained with 0.4% trypan blue solution (HyClone). After staining, the percentage of cell coverage of the culture wells was calculated using Fiji ImageJ [17].

### Assessment of senescence

AT-MSCs were seeded on a 6-well plate (Eppendorf) at the density of 4.0 × 10^3^ cells/cm^2^ in the culture medium (Table 1). The senescence of AT-MSCs was analyzed using Senescence β-Galactosidase Staining Kit (Cell Signaling Technology) according to the manufacturer's protocol. The cells were visualized using an Olympus IX81 microscope equipped with a MicroPublisher 3.3 RTV camera (Olympus).

### Collection of conditioned media from AT-MSC culture

The AT-MSCs were incubated in αMEM medium with 1% human serum albumin (Grifols) and 1% P/S solution for 24 h. The conditioned media were collected and stored at − 80 °C. On the day of analysis, the conditioned media were thawed at 37 °C and centrifuged to remove the remaining cells, cellular debris, and apoptotic bodies.

### Assessment of the pro-angiogenic properties of the AT-MSC secretome

#### Quantification of concentrations of human angiogenesis-related molecules, cytokines and growth factors in the conditioned media from AT-MSC culture

MILLIPLEX Human Angiogenesis/ Growth Factor Magnetic Bead Panel (Merck) containing beads for the following analytes: angiopoietin-2, endothelin-1, EGF, HB-EGF, VEGF-A, VEGF-D, leptin, follistatin, IL-8, FGF-1, FGF-2, HGF, PLGF, BMP-9, G-CSF was used to analyze the concentrations of the listed analytes in the conditioned media collected from AT-MSC cultures. The analysis was conducted using undiluted media according to the manufacturer’s protocol on the Luminex 200 Instrument System (Millipore, Merck).

#### Capillary-like tube formation assay

HUVECs (Lonza) were cultured in EGM-2MV Microvascular Endothelial Cell Growth Medium-2 BulletKit (Lonza). 24-well plate (Eppendorf) was coated with Matrigel Matrix Grow Factor Reduced (BD Pharmingen). The HUVECs were seeded at a density of 7.5 × 10^4^ cells/ well in the collected conditioned media (from AT-MSC culture). Tube formation was investigated with a Leica DMI6000B (ver. AF7000) microscope and analyzed with ImageJ software.

### Detection of the marker of oxidative stress- 8-hydroxy-2’-deoxyguanosine (8OHdG)

From AT-MSCs cultivated in the every type of culture medium (Table 1) total DNA was isolated using the GeneMATRIX Cell Culture DNA Purification Kit (EURx) according to the manufacturer's protocol. AT-MSCs treated with 10 ng/ml IL-1β (BioLegend) under hypoxia conditions (37 °C, 5% CO_2_, 5% O_2_) were used as a positive control. The concentration of 8OHdG in cells was determined using the DNA Damage Competitive ELISA Kit (Thermo Fisher Scientific) according to the manufacturer’s protocol. Absorbance was measured at 450 nm using the Multiscan FC plate reader.

### Adipogenic, osteogenic and chondrogenic differentiation of AT-MSCs

In the case of adipogenic and osteogenic differentiation, 1.9 × 10^4^ cells/ well were seeded on a 12-well plate (BD Falcon) in the culture medium (Table 1). To induce chondrogenic differentiation, micro mass cultures were generated by seeding 5 µl droplets of cell solution (one droplet contained 4 × 10^4^ cells) on a 12-well plate (BD Falcon) and flooded with standard CM or CM with addition of glucose (25 mM) or glucose (25 mM) and insulin (1 µg/ml) (Table 1). After 24 h, CM was replaced with differentiation medium (DM) listed in the Table 1, which were used in three variants: (i) without additional ingredients (control DM), or with the addition of (ii) 25 mM glucose, or (iii) 25 mM glucose and 1 µg/ml insulin. At 3, 7, 14, and 21 days, adipogenic, osteogenic, and chondrogenic differentiation was confirmed by (i) mRNA analysis, and (ii) histological staining.

### Gene expression analysis

Total RNA was isolated and reverse transcribed using the Universal RNA/ miRNA Purification Kit (EURx) and the NG dART RT Kit (EURx), respectively, according to the manufacturer’s protocol and using C1000 Touch ThermalCycler (Bio-Rad). The expression of genes related to adipogenesis (CEBPα, PPRγ), osteogenesis (osteocalcin, osteopontin, Runx2) and chondrogenesis (SOX9, ACAN, COL2A1, COL10A1, HPLN) was examined by real-time PCR using QuantStudio 6 Flex Real-Time PCR System (Thermo Fisher Scientific).

### Histological staining

To demonstrate the presence of lipid droplets characteristic of adipogenic differentiation, cells fixed with 4% paraformaldehyde (CHEMPUR) were incubated with 60% isopropanol (POCH) for 15 min and stained with 1% Oil Red O solution (Merck) for 15 min. In case of osteogenic differentiation, fixed cells were stained with 2% Alizarin Red S solution (Merck) to indicate calcium phosphate deposition. To evaluate the presence of chondroitin sulphate in differentiated chondrogenic culture, fixed cells were stained with Alcian Blue Staining Solution (Merck) for 30 min. After histological staining, cells were visualised using an Olympus IX81 microscope.

### Statistical analysis

Statistical analysis was performed using GraphPad Prism 8.4 software (Graph Pad Software). Data are presented as the mean ± SD or means (on heat maps). Each experiment was conducted in triplicate using three independent replicates. One unit include one cell sample derived from one type of AT-MSCs cultured in one type of medium. For experiments with Gaussian distribution results, two-way ANOVA followed by NIR-Fisher or Tukey’s multiple comparisons was used to determine the statistical significance. For non-parametric probes, the Kruskal Willis test with Uncorrected Dunn’s test as a post hoc test was used. For supplementary data, an unpaired *t*-test was used. Results were considered statistically significant when *p < 0.05; **p < 0.01; ***p < 0.001. Biostatistical consultation was performed to assess the number of biological replicates per group. The sample size was calculated using a formula adapted from the resource equation method described by Charan and Kantharia [18].

Considering the number of donors, cell types and culture conditions, the calculated E-value was 12, which falls within the recommended range of 10–20 for an appropriately designed study.

## Results

### Influence of type 2 diabetes on the biological properties of AT-MSCs

The experimental layout (Additional file 1, Fig. S1) was designed to compare the biological properties of human AT-MSCs from healthy and T2D donors cultured under standard culture conditions (CM) or culture conditions that mimic the diabetic tissue microenvironment of patients treated (or not) with insulin in vitro. It was also designed to investigate the possibility of developing ATMPs containing AT-MSCs from diabetic adipose tissue donors and to evaluate the potency of diabetic AT-MSCs.

**Fig. 1:**
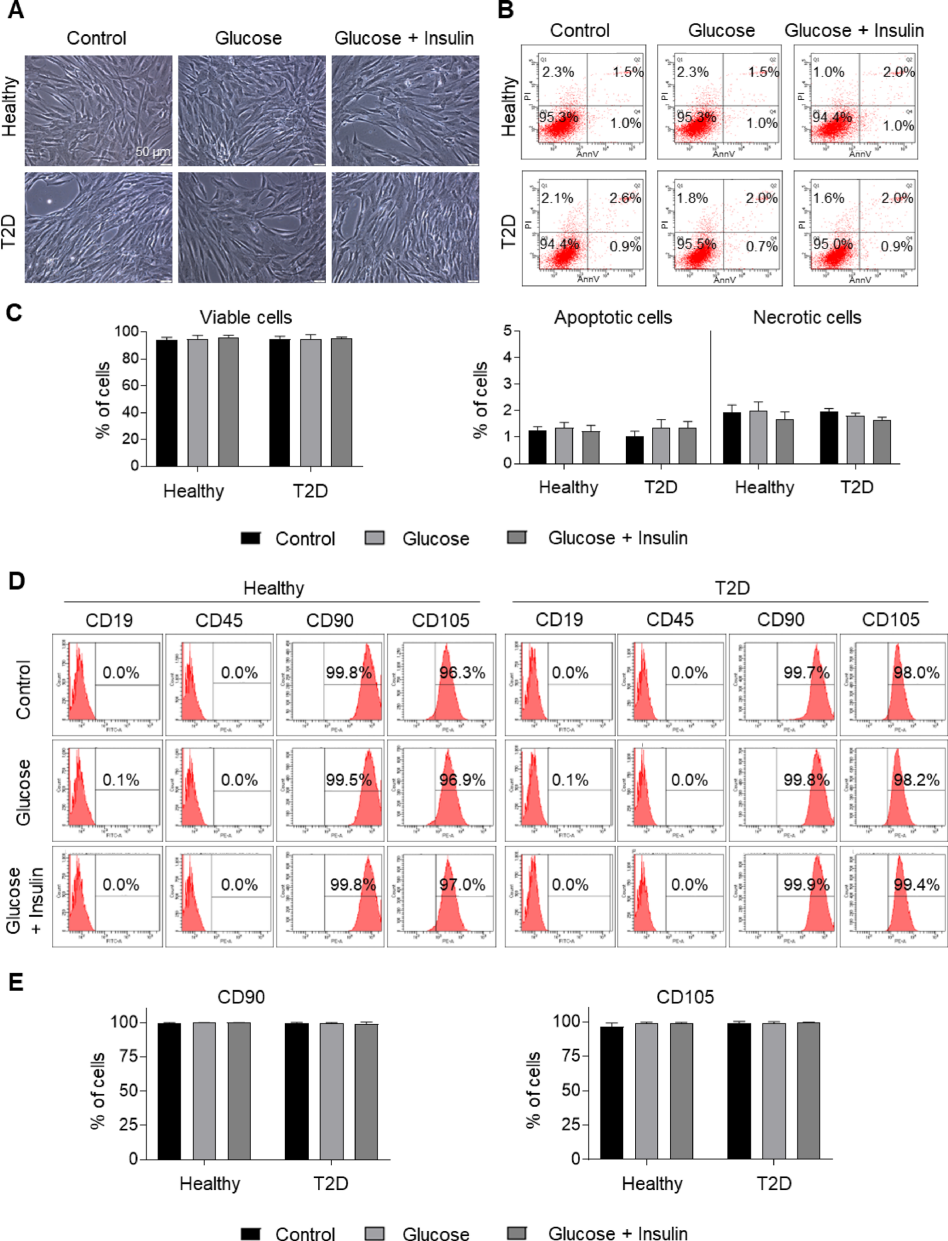
Biological characterization of AT-MSCs from healthy and T2D donors. AT-MSCs were cultured in the standard CM (Control), CM with addition of 25 mM glucose (Glucose), or CM with addition of 25 mM glucose and 1 µg/ml insulin (Glucose + Insulin). **A** Morphology of AT-MSCs. Scale bars: 50 µm. **B** Representative dot-plots from analysis of viability of both types of AT-MSCs by flow cytometry. Cells were stained with Annexin V Apoptosis Detection Kit and analyzed by BD LSR Fortessa flow cytometer and FACS Diva Software. **C** Quantitative data representing the percentage of viable (AnnV^−^/PI^−^), apoptotic (AnnV^+^/PI^−^, AnnV^+^/PI^+^) or necrotic (AnnV^−^/PI^+^) AT-MSCs. **D** Antigenic profile of AT-MSCs by flow cytometry. Representative histograms of expression of selected MSC-negative markers (CD19, CD45) and MSC-positive markers (CD90, CD105) on both types of AT-MSCs. **E** Quantitative data representing the percentage content of AT-MSCs positive for analyzed antigens. Results are presented as mean ± SD, n = 3 (biological replicates). *AnnV* annexin V; PI Propidium iodide; Healthy – Healthy donor; T2D Diabetic donor

Both AT-MSC populations were cultured in αMEM medium containing 5% hPL (intended for MSC culture for further clinical applications) supplemented (or not) with glucose or glucose + insulin. AT-MSCs from healthy and T2D donors exhibited a spindle-shaped, fibroblast-like cell morphology characteristic of MSCs in all used media (Fig. 1A).

The viability of both AT-MSC populations was higher than 94% with a low content of apoptotic and necrotic cells, independently of the type of culture medium (Fig. 1B, C). Furthermore, AT-MSCs from healthy and diabetic adipose tissue donors showed comparable expression of selected MSC-specific markers such as CD105 and CD90 (> 95% positive cells) and in parallel did not express markers of hematopoietic cells such as CD19 and CD45 (< 2% positive cells; Fig. 1D and E; Additional file 1, Fig. S2A). Interestingly, the density of CD90 antigen was significantly lower in AT-MSCs derived from T2D donors and independent of the used culture medium (Additional file 1, Fig. S2B).

**Fig. 2:**
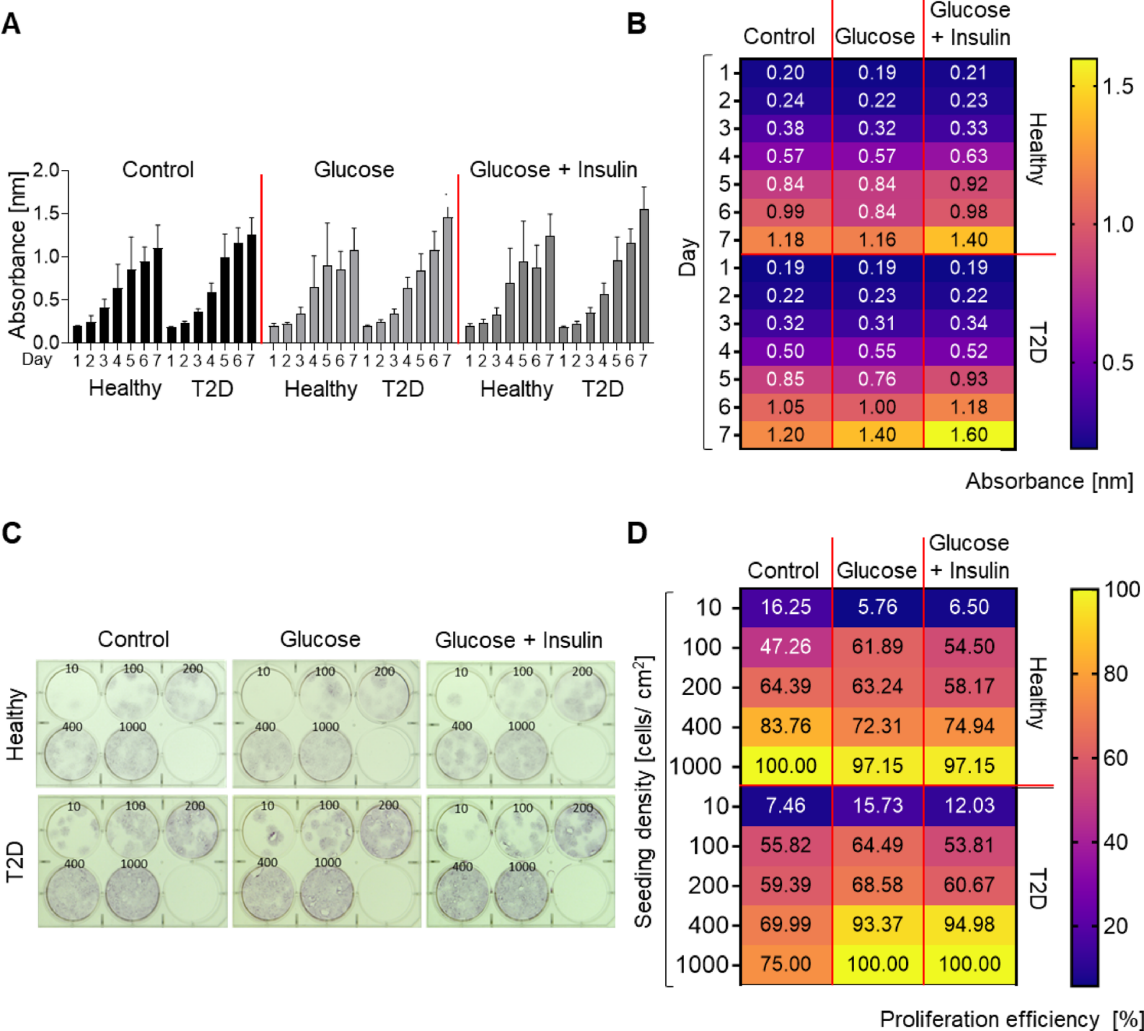
Proliferation capacity of AT-MSCs from healthy and T2D donors. AT-MSCs were cultured in standard CM (Control), CM with the addition of 25 mM glucose (Glucose), or CM with the addition of 25 mM glucose and 1 µg/ml insulin (Glucose + Insulin). **A** Proliferation rate of AT-MSCs by a colorimetric assay using the Cell Counting Kit-8. Higher absorbance values correlate with higher proliferation rate. Results are presented as mean ± SD, n = 3; Kruskal Willis test with Uncorrected Dunn’s test as post hoc test was used for statistical analysis. **B** Heat map of the proliferation of AT-MSCs. The results are presented as means, n = 3. **C** Representative images of proliferation efficiency (calculated as the percentage of culture well coverage by AT-MSCs). The AT-MSCs were seeded at densities of 10, 100, 200, 400, 1000 cells/well (9.5 cm^2^) in the tested media and after 21 days stained with trypan blue solution. **D** Quantitative analysis of AT-MSC proliferation efficiency presented as percentage of culture well coverage by AT-MSCs. Results are presented as mean, n = 3 (biological replicates); Kruskal Willis test with Uncorrected Dunn’s test as post hoc. Healthy – healthy donor; T2D – diabetic donor

AT-MSCs from healthy and T2D donors cultured in the standard culture medium (CM, Control) or CM additionally supplemented with glucose or glucose + insulin, showed a similarly high proliferation rate over the consecutive 7 days of culture. Furthermore, diabetic AT-MSCs in CM supplemented with glucose or glucose + insulin after 6–7 days of culture showed the highest proliferation rate when compared to AT-MSCs from healthy donors (Fig. 2A, 2). To confirm the observed high proliferation efficiency of the cells, both subpopulations of AT-MSCs were seeded at different concentrations (per well) and subsequently after 21 days, the percentage of culture well coverage by seeded cells was calculated (Fig. 2C). Our analysis confirmed the high proliferation efficiency of both types of AT-MSCs. However, AT-MSCs from healthy donors and cultured in the standard CM showed a higher tendency to cover the well when compared to AT-MSCs from T2D donors in the same culture conditions.

When cultured in CM supplemented with glucose and glucose + insulin, diabetic AT-MSCs had a higher tendency to cover the wells (when compared to AT-MSCs from healthy donors) (Fig. 2C, D). Statistical analysis revealed no differences in proliferation efficiency between both types of AT-MSCs independently of the additional supplementation of CM (Fig. 2D). The obtained results confirmed the possibility of expanding both subpopulations of AT-MSCs to manufacture a given dose of cell-based ATMPs. Furthermore, only single (large area) senescent β-galactosidase-positive (blue-stained) AT-MSCs from healthy and T2D donors were observed per microscopic field in the tested media (Additional file 1, Fig. S2C). The AT-MSCs used in the current study were maintained in good condition through 4th to 6th passages, regardless of the presence of T2D in the tissue donors and the type of used culture medium. 8OHdG, a marker of oxidative stress, was not detected in either subpopulation of AT-MSCs cultured in standard CM. The concentration of 8OHdG in AT-MSCs from healthy donors subsequently treated with glucose or glucose + insulin was higher than in diabetic AT-MSCs (under the same culture conditions). Supplementation of culture media with glucose or glucose + insulin did not increase 8OHdG levels in T2D donor-derived AT-MSCs (Additional file 1, Fig. S3A). These findings may be an indication that the application of AT-MSCs from healthy donors to T2D recipients may induce their oxidative stress and support autologous cell administration.

**Fig. 3:**
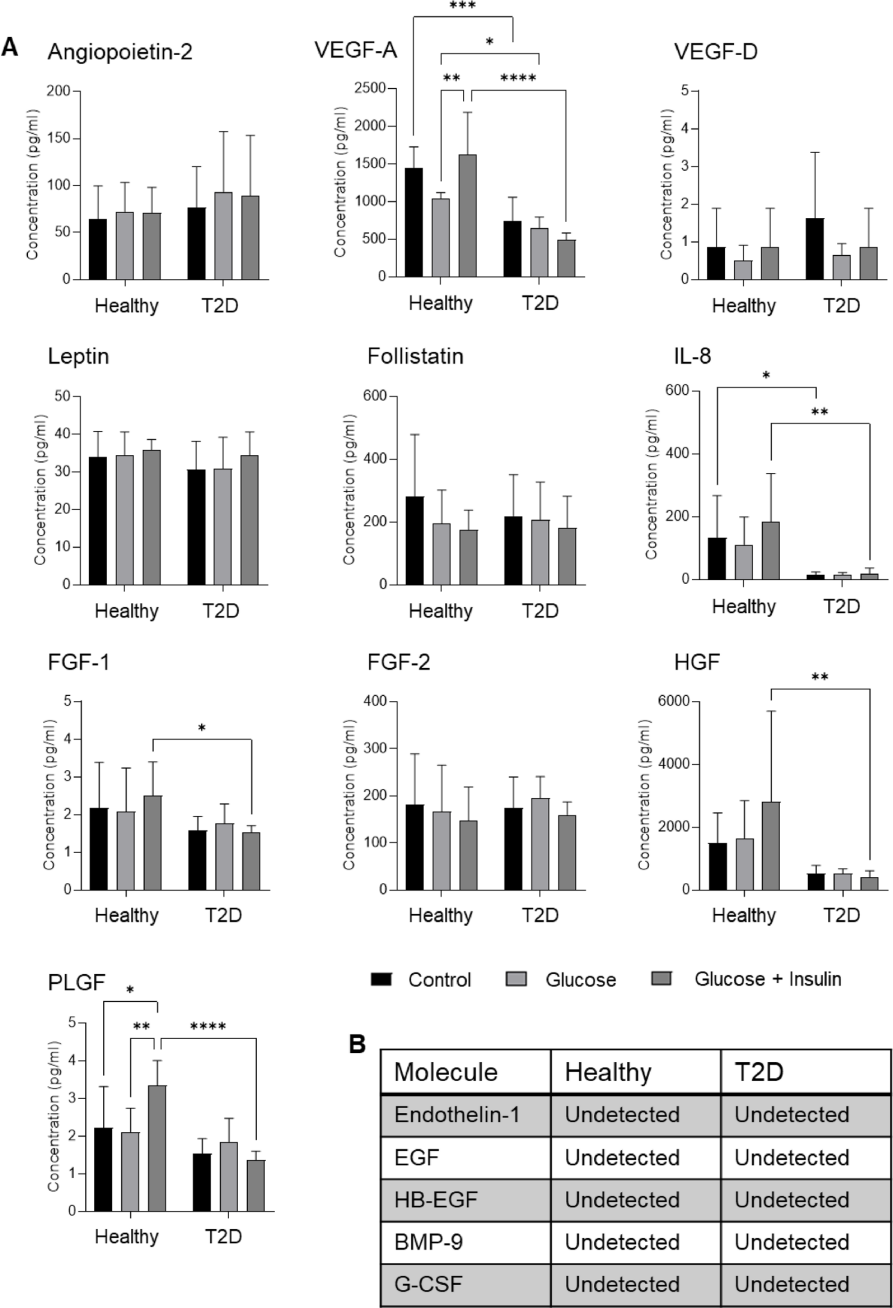
Secretion of angiogenesis-related molecules and growth factors by AT-MSCs. **A** Concentration of selected angiogenesis-related molecules and growth factors in the conditioned media collected from healthy and diabetic AT-MSC culture. The AT-MSCs were cultured in the following media: standard CM (Control), CM with 25 mM glucose (Glucose) or CM with 25 mM glucose and 1 µg/ml insulin (Glucose + Insulin), and subsequently the listed media were replaced with αMEM supplemented with 1% HSA (for 24 h). To assess the concentration of analyzed molecules, MILLIPLEX Multiplex Assay using Luminex technology was used. Results are presented as mean ± SD, n = 3 (biological replicates); two-way Anova with Tukey’s multiple comparisons test. **B** Analytes not detected in AT-MSC-derived conditioned media. Healthy – healthy donor; T2D – diabetic donor

As MSCs exhibit widespread paracrine activity, we subsequently analyzed the concentration of selected growth factors and angiogenesis-related molecules in the conditioned media derived from AT-MSC cultures (Fig. 3). We observed comparable concentrations of angiopoietin-2, VEGF-D, leptin, follistatin, FGF-1, FGF-2, HGF and PLGF in the conditioned media of healthy and diabetic AT-MSC cultures that had previously been cultured in the standard CM.

Adding glucose, or a combination of glucose and insulin, to CM does not significantly impact the secretion of the majority of analyzed molecules by either AT-MSC subpopulation. However, the addition of glucose and insuline effects the secretion of VEGF-A and PLGF by AT-MSCs derived from healthy donors. The analysis also revealed that healthy AT-MSCs secrete a significantly higher amount of VEGF-A and the pro-inflammatory cytokine IL-8 than cells from diabetic patients (Fig. 3A). Secretion of endothelin-1, EGF, HB-EGF, BMP-9 or G-CSF was not observed in either population of AT-MSCs (Fig. 3B).

**Fig. 4:**
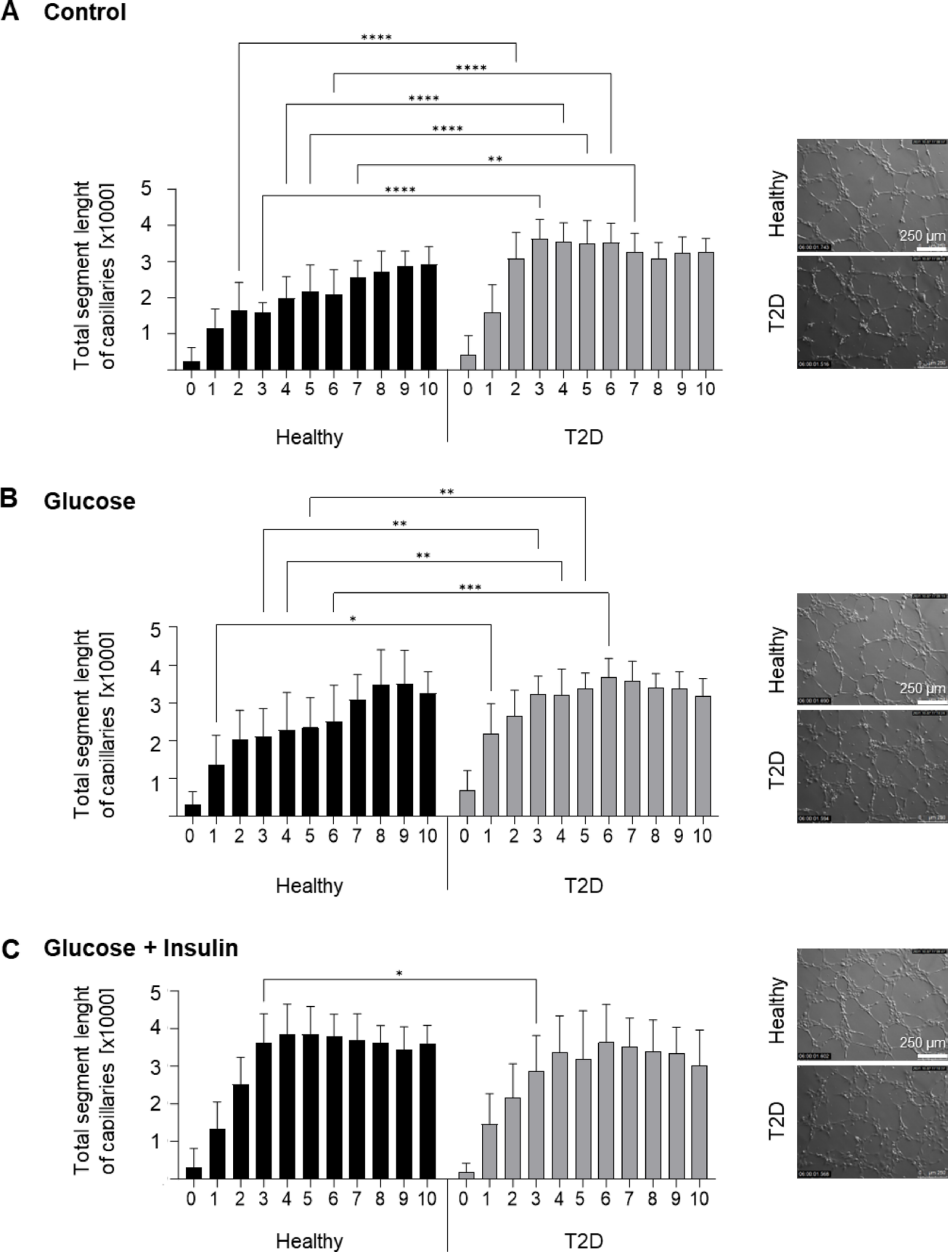
Analysis of the pro-angiogenic potential of the AT-MSC secretome. The AT-MSCs were cultured in standard CM (Control; (**A**)), CM with 25 mM glucose addition (Glucose; (**B**)) or CM with 25 mM glucose and 1 µg/ml insulin (Glucose + Insulin; (**C**)). To assess the pro-angiogenic properties of the AT-MSC secretome, HUVECs were seeded in Matrigel matrix-coated wells in the collected conditioned media (derived from the AT-MSC culture). Quantitative analysis of the pro-angiogenic potential of the AT-MSC secretome was presented as the total length of the capillaries per microscopic field at different time points, from the start of the assay (0 h) to 10 h. Results are presented as mean ± SD, n = 3 (biological replicates); two-way Anova with NIR Fisher test as post hoc. On (a-c) panels, representative bright-field images of HUVECs incubated in the conditioned media derived from culture of both types of AT-MSCs were presented at 6 h of the assay. Scale bars: 250 µm. Healthy – healthy donor; T2D – diabetic donor

Since angiogenesis is one of the crucial steps in the regeneration of injured tissues, *e.g.* in cases of ischemic tissue injury [19] or bone regeneration which is centered on the interaction between osteogenic and angiogenic events [20], we investigated whether the conditioned media collected from both types of AT-MSC culture might stimulate the pro-angiogenic potential of HUVECs, i.e. their ability to form capillary-like structures (Fig. 4A–C). The greatest pro-angiogenic potential was observed in HUVECs incubated with conditioned media derived from diabetic AT-MSCs and was independent of supplementation of the diabetic AT-MSC culture medium with glucose or glucose + insulin (Additional file 1, Fig. S3B). HUVECs incubated in conditioned CM derived from healthy donor AT-MSCs showed lower pro-angiogenic potential when compared to HUVECs incubated in conditioned CM derived from diabetic AT-MSC culture (Fig. 4A). Interestingly, the addition of glucose + insulin to the medium for healthy donor-derived AT-MSCs can positively stimulate the capillary-like tube formation by HUVECs (Fig. 4B, C).

In conclusion, diabetic AT-MSCs showed a morphology characteristic of MSCs, a high proliferation rate, viability and a low content of senescent cells under standard culture conditions in vitro. In addition, diabetic AT-MSCs exhibited a comparable ability to secrete angiogenesis-related molecules and growth factors such as angiopoietin-2, VEGF-D, leptin, follistatin, FGF-1, FGF-2, HGF and PLGF as cells from healthy donors along with lower secretion of VEGF-A and IL-8. Furthermore, the conditioned medium from the culture of diabetic AT-MSCs is more effective at stimulating HUVECs to form capillary-like structures than the medium from healthy patient cells. This suggests that molecules/particles other than those examined, such as microRNAs, chemokines, or EVs, may also be involved. Neither healthy nor diabetic AT-MSCs express markers of oxidative stress in the standard culture conditions. However, transplantation of AT-MSCs from healthy donors into T2D recipients may induce their oxidative stress and, on the other hand, stimulate their secretion of pro-angiogenic factors.

### Adipogenic, chondrogenic and osteogenic differentiation of AT-MSCs from healthy and diabetic donors

The potential for trilinear differentiation is one of the most widely recognized characteristics of MSCs [8]*.* In addition, the ability of MSCs to directly differentiate into cells that form specific tissues is one of the mechanisms of their pro-regenerative activity [21]. Therefore, in the second phase of the study, the AT-MSCs from healthy and T2D donors were differentiated into adipocytes, chondroblasts, and osteoblasts in tissue-specific differentiation media, which were supplemented (or not) with glucose or glucose + insulin. Importantly, the baseline levels of the analyzed genes was comparable in both AT-MSC populations prior to the onset of differentiation (day 0) (Additional file 1, Fig. S4-6), except for Runx2 and COL10A1, whose expression was significantly lower in diabetic AT-MSCs along with higher HPLN expression.

**Fig. 5:**
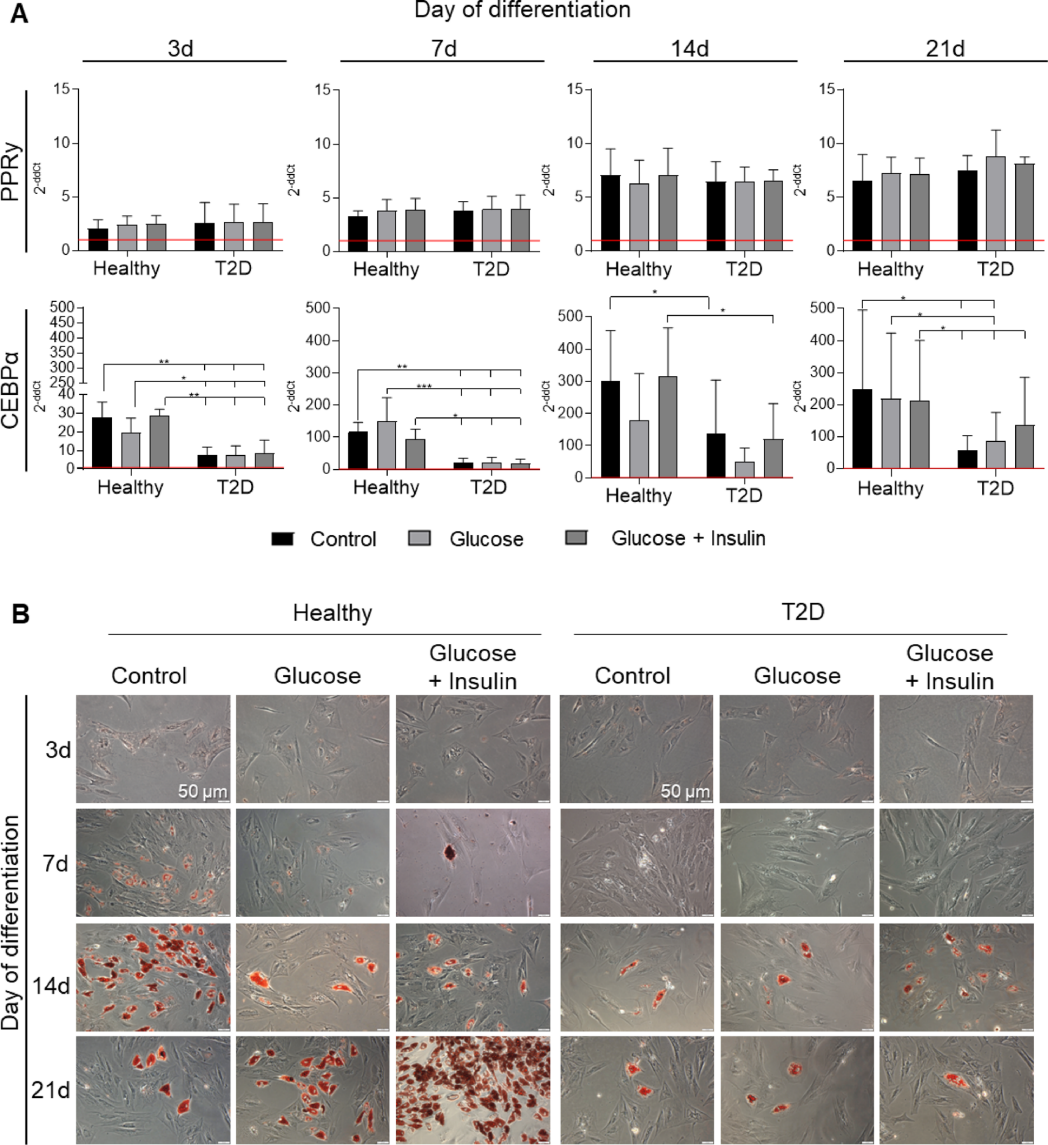
Adipogenic differentiation of AT-MSCs from healthy and T2D donors. The AT-MSCs were cultured in standard adipogenesis stimulating medium—StemPro Adipogenesis Differentiation Kit (Adipo-DM; Control), Adipo-DM with the addition of 25 mM glucose (Glucose) or Adipo-DM with the addition of 25 mM glucose and 1 µg/ml insulin (Glucose + Insulin) for 3, 7, 14 and 21 days. **A** Expression of adipogenesis-related genes (PPR-γ and CEBPα). Fold differences in expression (2^−ddCt^) of analyzed genes in undifferentiated cells (at day 0) separately for healthy- and T2D donors-derived AT-MSCs were calculated as 1.0 and marked by a solid red line. Results are presented as mean ± SD, n = 3 (biological replicates); two-way Anova with NIR Fisher test as post hoc was used. *p < 0.05; **p < 0.01; ***p < 0.001. **B** Oil Red O staining of AT-MSCs differentiated into adipocytes. Fixed AT-MSCs were stained with Oil Red O. Brownish-red oil droplets are characteristic of adipogenic differentiation. Scale bars: 50 µm. PPR-γ, peroxisome proliferator-activated receptor γ; CEBPα, CCAAT/enhancer-binding protein α. Healthy – healthy donor; T2D – diabetic donor

In the case of adipogenic differentiation, the mRNA expression of the adipogenesis-related transcription factor PPARγ was comparable in AT-MSCs from healthy and T2D donors on consecutive days of differentiation (Fig. 5A). We also observed significantly decreased expression of another adipogenesis-related gene– CEBPα–in diabetic AT-MSCs at each analyzed time point (3, 7, 14 or 21 days) when compared to AT-MSCs from healthy donors. Furthermore, supplementation of the differentiation media with glucose or glucose + insulin had no significant effect on the expression of the analyzed genes (Fig. 5A). Histological staining revealed reduced aggregation of lipid droplets characteristic of adipogenic differentiation in diabetic AT-MSCs (Fig. 5B), suggesting impaired adipogenesis in such population of AT-MSCs.

**Fig. 6:**
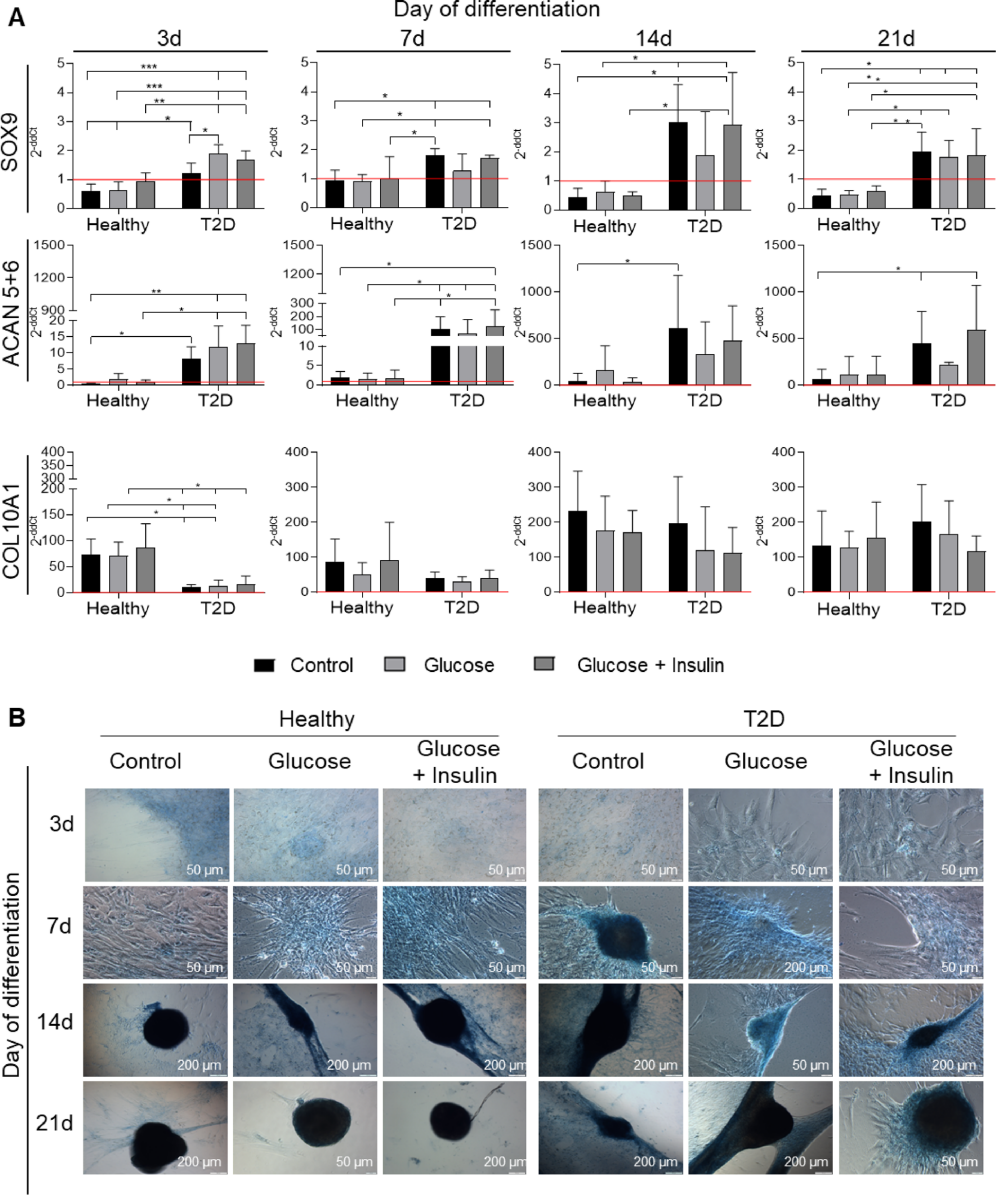
Chondrogenic differentiation of AT-MSCs from healthy and T2D donors. The AT-MSCs were cultured in standard chondrogenesis stimulating medium StemPro Chondrogenesis Differentiation Kit (Chondro-DM, Control), Chondro-DM with the addition of 25 mM glucose (Glucose), or Chondro-DM with the addition of 25 mM glucose and 1 µg/ml insulin (Glucose + Insulin) for 3, 7, 14 and 21 days. **A** Expression of chondrogenesis related genes (SOX9, ACAN5 + 6 and COL10A1). Fold differences in expression (2^−ddCt^) of analyzed genes in undifferentiated cells (at day 0) separately for healthy- and T2D donors-derived AT-MSCs were calculated as 1.0 and marked by a solid red line. Results are presented as mean ± SD, n = 3 (biological replicates); two-way Anova with NIR Fisher test as post hoc for SOX9; Kruskal Willis test with Uncorrected Dunn’s test as post hoc test for ACAN 5 + 6 and COL10A1 were used for statistical analysis. *p < 0.05; **p < 0.01; ***p < 0.001. **B** Alcian Blue staining of AT-MSCs differentiated into chondroblasts. Fixed AT-MSCs were stained with Alcian Blue. Blue staining of sulphated proteoglycans is characteristic of chondrogenic differentiation. Scale bars: 50 µm or 200 µm (as indicated). Sox9, key transcription factor in early chondrogenesis; ACAN 5 + 6, aggrecan 5 and 6; COL10A1, collagen type X alpha 1 chain. Healthy – healthy donor; T2D – T2D donor

Focusing on chondrogenic stimulation, we confirmed that both AT-MSC populations had the ability to undergo chondrogenic differentiation (Fig. 6A, B). However, a higher expression of chondrogenesis-related genes—Sox9, ACAN 5+ 6—was observed in diabetic AT-MSCs when compared to AT-MSCs from healthy donors at each experimental time point (Fig. 6A). The fold change in COL2A1 expression was also slightly increased in AT-MSCs from T2D donors, whereas we did not observe any significant change in HPLN gene expression between the analyzed AT-MSCs (Additional file 1, Fig. S7). Importantly, the expression of COL10A1 (which may be involved in chondrocyte hypertrophy) was higher in AT-MSCs from healthy donors after 3 days of differentiation and subsequently reached comparable levels in both populations of AT-MSCs (Fig. 6A). Histological staining of AT-MSCs differentiated into chondroblasts showed extracellular secretion of sulphated proteoglycans (characteristic of chondrogenic differentiation) by both AT-MSC populations, and differentiation kinetics appeared to be faster in diabetic AT-MSCs (Fig. 6B). Supplementation of the differentiation medium with glucose or glucose + insulin did not significantly affect the chondrogenic differentiation of either AT-MSC population (Fig. 6A, B).

**Fig. 7:**
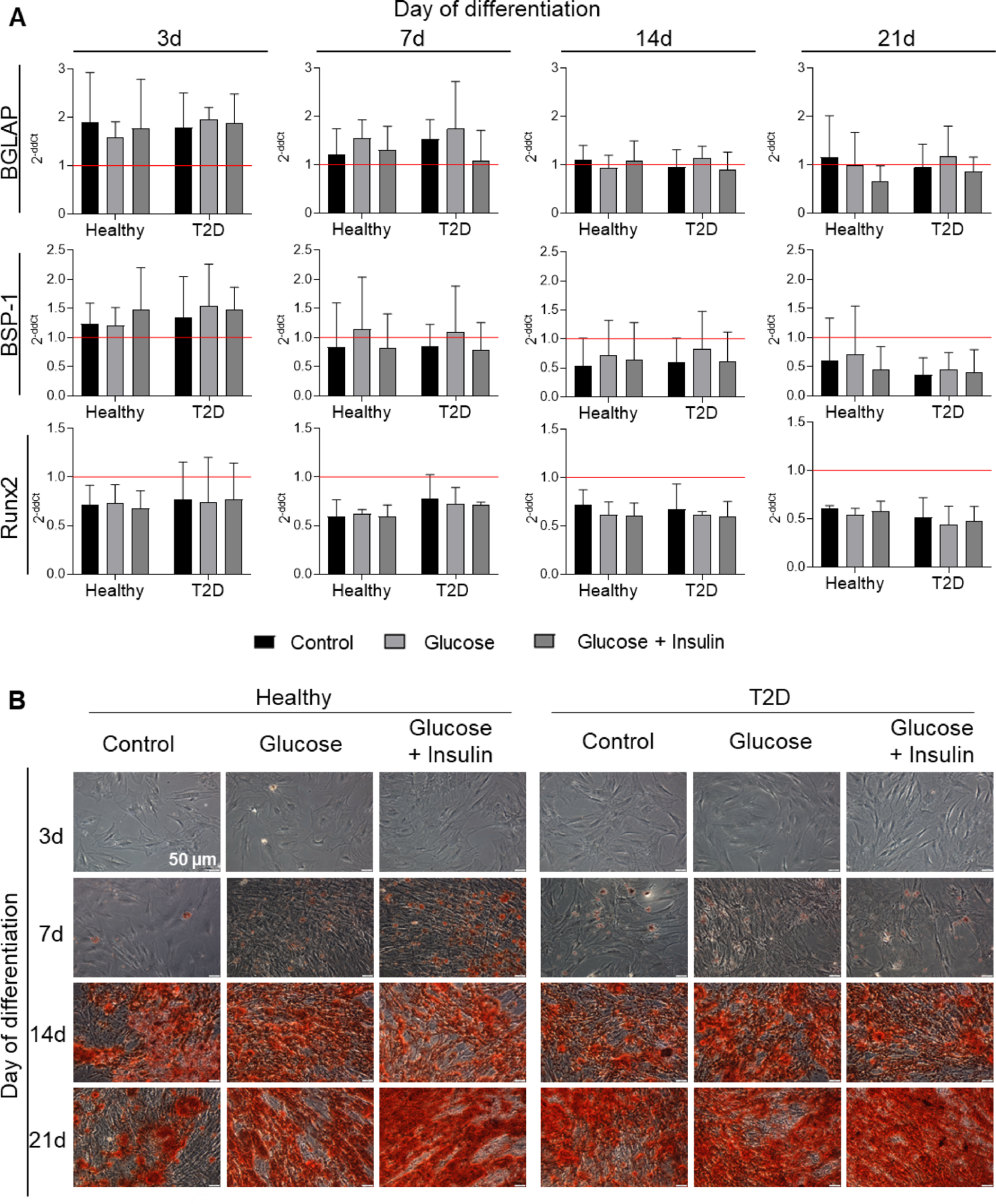
Osteogenic differentiation of AT-MSCs from healthy and T2D donors. AT-MSCs were cultured in the standard osteogenesis stimulating medium- StemPro Osteogenesis Differentiation Kit (Osteo-DM; Control) or Osteo-DM with addition of 25 mM glucose (Glucose), iii) or Osteo-DM with addition of 25 mM glucose and 1 µg/ml insulin (Glucose + Insulin) for 3, 7, 14 and 21 days. **A** Expression of osteogenesis-related genes (BGLAP, BSP-1 and Runx2). Fold differences in expression (2^−ddCt^) of analyzed genes in undifferentiated cells (at day 0) separately for healthy- and T2D donors-derived AT-MSCs were calculated as 1.0 and marked by a solid red line. The results are presented as mean ± SD, n = 3 (biological replicates); two-way Anova with the NIR Fisher test as post hoc was used for statistical analysis. *p < 0.05; **p < 0.01; ***p < 0.001. **B** Alizarin Red S staining of AT-MSCs differentiated into osteoblasts. Fixed AT-MSCs were stained with Alizarin Red S. Red staining of calcium phosphate deposits is characteristic of osteogenic differentiation. Scale bars: 50 µm. BGLAP, bone gamma-carboxyglutamate protein; BSP-1, bone sialoprotein 1; Runx2, runt-related transcription factor 2 (a transcription factor closely associated with the osteoblast phenotype). Healthy – healthy donor; T2D – diabetic donor

In the case of osteogenic differentiation, no significant differences in the expression of osteogenesis-related genes like Runx2, BGLAP, BSP-1 were observed at the analyzed time points (3, 7, 14 or 21 days of differentiation) between both types of AT-MSCs when compared to undifferentiated AT-MSCs, independently of additional supplementation of differentiation medium. The slightly increased expression of mRNA for the BGLAP and BSP-1 genes was observed mainly after 3 days of differentiation in both AT-MSC populations (Fig. 7A). Histological staining of both types of AT-MSC showed a comparable deposition of red colored calcium phosphates characteristic of osteogenic differentiation (Fig. 7B), indicating that T2D does not affect AT-MSC osteogenesis.

In conclusion, the obtained results confirm that diabetic AT-MSCs have a lower adipogenic differentiation potential, a higher chondrogenic differentiation capacity and a comparable osteogenic differentiation capacity when compared to AT-MSCs from healthy donors. Thus, tissues from both types of donors can be used to develop AT-MSC-based ATMPs.

## Discussion

The development of a cell-based ATMP intended for the treatment of selected tissue injuries that will have a beneficial effect for the patient is a real scientific challenge and requires the performance of both basic and advanced research, as well as the implementation of a process approach to establish the manufacturing process [22]. The first step in the development of a cell-based ATMP is to define the criteria for the inclusion and exclusion of donors from whom tissue will be collected as the starting material for further ATMP manufacturing.

In the current study, we compared the biological potential of AT-MSCs derived from healthy and diabetic tissue donors to answer the question of the possibility of developing ATMPs specifically from diabetic donor-derived adipose tissue, whose properties are changed during diabetes [23]. Because MSCs interact with their microenvironment and are stimulated by the microenvironmental factors [9], for the first time the biological properties of AT-MSCs were investigated in different tissue microenvironments (in vitro*)* that mimic diabetic conditions in donors/recipients treated (or not) with insulin. It allowed us to evaluate the biological potential of diabetic AT-MSCs in in vitro research for further clinical applications, given that some researchers have reported abnormal characteristics or dysfunction of diabetic MSCs, which may limit their autologous use, as indicated by Kornicka et al*.*, among others, based on the systematic review of the available literature [14]​. Nagaishi et al*.* demonstrated abnormal morphology and proliferative capacity along with increased endoplasmic reticulum stress of bone marrow-derived MSCs (BM-MSCs) isolated from streptozotocin-induced diabetic rats, which resulted in loss of their therapeutic effects in vivo [13]. The impaired proliferation and angiogenic activities of rat diabetic BM-MSCs and their ineffectiveness for repairing tissues during hindlimb ischemia in vivo has been also presented [24, 25]. Moreover, the reduced number of BM-MSCs in diabetic patients and streptozotocin-induced diabetic rats was also observed [15, 26] along with their aberrant osteogenic and angiogenic differentiation capacities [25, 26]. The above reports clearly demonstrate the necessity of the research presented in this work, which may have a significant impact on patient outcomes following MSC therapy.

**Fig. 8:**
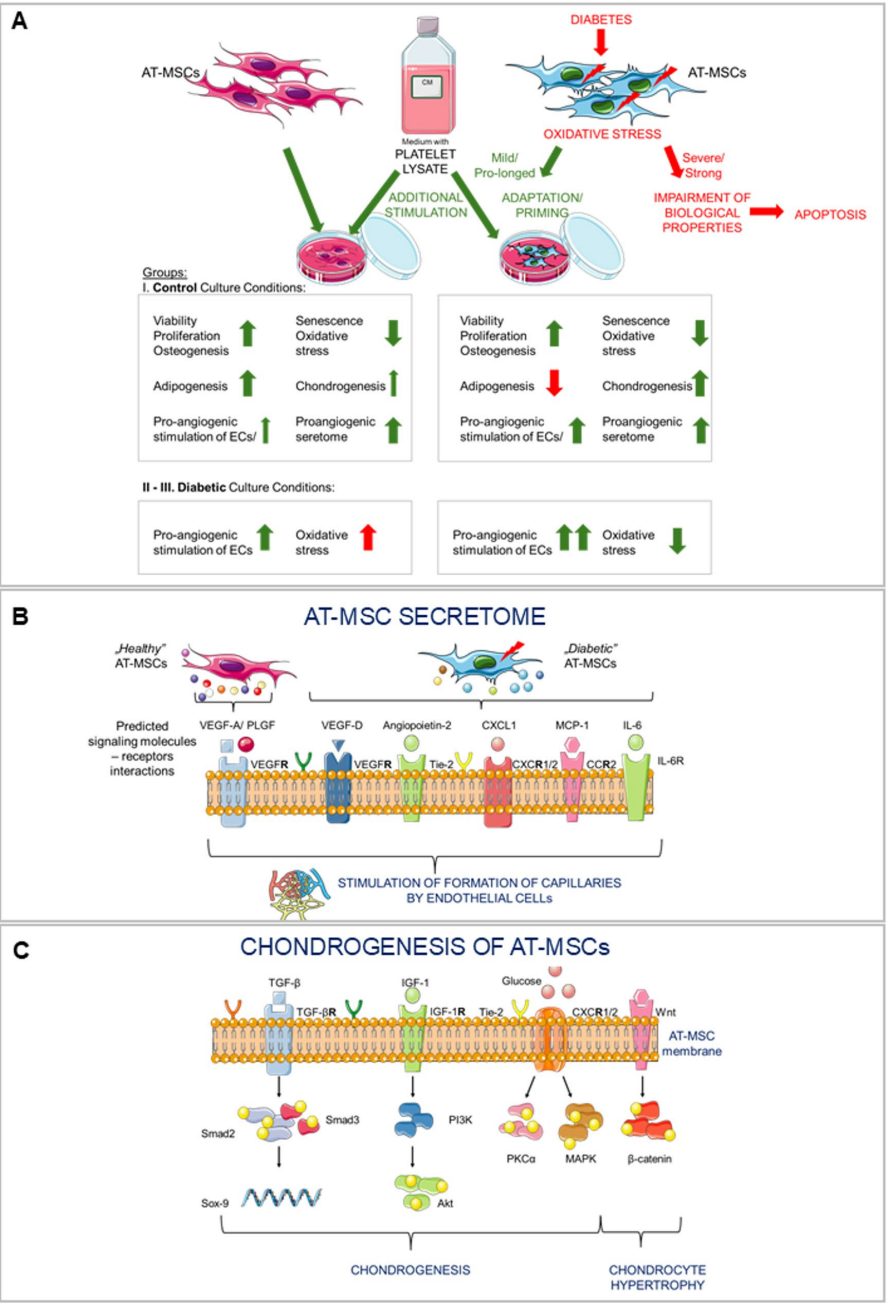
Mechanistic overview of the biological properties of AT-MSCs from healthy and diabetic donors. **A** Summary of the biological properties of both populations of AT-MSCs including predicted impact of diabetes-induced oxidative stress and human platelet lysate (hPL) stimulation. **B** Predicted possible signalling “molecule – receptor” interactions that may stimulate pro-angiogenic capacity of AT-MSCs and formation of capillary-like structures, based on the factors secreted by AT-MSCs and present in hPL used as supplement for AT-MSC culture. **C** Signalling pathways possibly involved in chondrogenesis of AT-MSCs, stimulated by factors present in hPL

The study used AT-MSCs, which have several advantages over BM-MSCs. These include a higher rate of cell proliferation and a greater anti-apoptotic and antioxidant capacity [27]. AT-MSCs from both healthy and T2D tissue donors exhibited morphology characteristic of MSCs, high viability, and comparable expression of selected MSC markers during ex vivo expansion, independently of additional supplementation of the culture medium (Fig. 8A). However, the surface density of CD90 and CD105 antigens was slightly higher in healthy donor-derived AT-MSCs compared to diabetic AT-MSCs, which may indicate their greater osteogenic potential as shown by Chung et al*.* [28]. In addition, both populations of AT-MSCs showed a comparable high proliferation rate and a low content of senescent cells, which are important parameters during the cultivation of cells for the production of cell-based ATMPs. Interestingly, we observed that AT-MSCs cultured in basal medium supplemented with fetal bovine serum (FBS) did not proliferate after the 3rd or 4th passage and only increased their surface area (data not shown). Therefore, AT-MSCs were cultured in αMEM medium supplemented with 5% hPL dedicated to MSC culture for further clinical applications. This allowed us to culture AT-MSCs for a much longer period than would have been possible using culture medium supplemented with FBS. It has been demonstrated that hPL contains high concentrations of proteins and growth factors, including PDGF-AA, PDGF-AB, VEGF, IGF-1, TGF-β, EGF and bFGF, which were undetected in FBS [29]. These growth factors may promote the proliferation, cell cycle progression and migration of human umbilical cord MSCs by upregulating relevant genes and proteins, and by activating Beclin1-dependent autophagy via the AMPK/mTOR signaling pathway [30]. Furthermore, EVs secreted by MSCs in a culture medium supplemented with hPL are enriched in molecules related to angiogenesis and promote angiogenic events in vitro and in vivo, compared to EVs from MSC culture medium supplemented with FBS [31]. For comparison, the investigators reported abnormal biological properties in human and rat diabetic MSCs, which were mainly cultured using a medium supplemented with FBS [13, 24, 25]. To the best of our knowledge, no scientific report has shown that diabetic MSCs cultured in a medium supplemented with hPL exhibit impaired biological potential. Thus, we postulate that hPL had positive significant impact on biological properties of AT-MSCs (or “primed” them) in the current study. It is particularly important for diabetic AT-MSCs.

8OHdG, a marker of oxidative stress, was undetected in AT-MSCs from either type of donor. However, high glucose concentrations and the presence of insulin increased 8OHdG levels in AT-MSCs from healthy donors, suggesting that allogeneic administration of AT-MSCs from healthy donors to T2D recipients taking insulin may induce their oxidative stress. Although oxidative stress marker was presented in these cells, the stress did not impact the cells' biological properties including *e.g.* viability, proliferation or differentiation potential. According to the oxidative stress concept published by Lushchak et al*.* [32], such oxidative stress can be classified as mild—does not impact cell viability. The other type of stress—strong—may be divided into two categories: (i) severe oxidative stress, which induces cell damage, and (ii) strong oxidative stress, which induces cell damage and cell death [32]. Researchers usually only describe the effects of oxidative stress on cells, rather than its severity, that impact on cell condition. For example, it has been demonstrated that exposing MSCs to oxidative stress significantly decreases their proliferation and metabolic activity, increases the release of proinflammatory cytokines, and reduces their migratory capacity in a wound closure assay [33]. Furthermore, oxidative stress in Wharton Jelly MSCs disrupts genomic stability and mitochondrial function, as well as affect transcription factor activity [34]. In AT-MSCs, it decreases the expression of stemness-associated genes, along with favouring the occurrence of phenotypes associated with inflammation and senescence [35]. Oxidative stress may also induce apoptosis, impair MSC differentiation capacity and limits the efficacy of their pro-regenerative potential [36, 37]. Importantly, it has been also demonstrated that AT-MSCs exposed to long-term oxidative stress activate an adaptive mechanism that restores their functional properties under stressful conditions [33]. However, this mechanism is currently unknown. We can postulate that diabetic AT-MSCs used in the current study, activated their adaptive mechanisms in response to long-term oxidative stress caused by long lasting diabetes in adipose tissue donors. This may explain their proper biological properties observed in the research. Because strong oxidative stress may induce cell apoptosis, the only way to eliminate the potential risks associated with allogeneic therapy using “healthy” AT-MSCs, which experience oxidative stress in the diabetic microenvironment, is to use an autologous ATMP. The other advantages of autologous ATMPs are that they are manufactured using the patient's own tissue and do not carry any risk of immune rejection, graft-versus-host disease development or transmission of infectious agents [38].

MSCs, besides direct differentiation into cardiomyocytes and vascular endothelial cells, can act through paracrine pathways to trigger angiogenesis, which is the main therapeutic mechanism of cell therapy for cardiovascular diseases [39]. We demonstrated that both types of AT-MSCs secrete angiopoietin-2, VEGF-D, leptin, follistatin, FGF-1, FGF-2, HGF and PLGF at the similar level. The similar secretion of VEGF, FGF-2, angiopoietin 2 and HGF was also observed between human healthy and diabetic MSCs from the bone marrow of the amputated limb. Although the investigators did not include information about the duration of diabetes in these patients, which may impact the severity of diabetes-induced oxidative stress [40], we can assume that the diabetes has been presented for a long time and was strong. Both types of AT-MSCs also secrete VEGF-A, which confirms their potency [41]. However, the secretion of VEGF-A by healthy AT-MSCs was significantly higher when compared to diabetic AT-MSCs. VEGF-A and its two tyrosine kinase receptors, VEGFR1 and VEGFR2, represent a key signalling pathway mediating physiological angiogenesis [42]. The VEGF family also includes PLGF, the concentration of which was higher in the conditioned medium collected from the culture of healthy AT-MSCs. Taken together, the pro-angiogenic activity of healthy AT-MSC secretome may result from VEGF-A/PLGF-VEGFR interactions, whereas in the case of diabetic AT-MSCs, it may result from VEGF-D-VEGFR or angiopoietin-2-Tie-2 interactions. However, we have also found that diabetic MSCs promote angiogenesis through unique secretome signatures with increased levels of chemokine (C-X-C motif) ligand 1 (CXCL-1), IL-6, and monocyte chemoattractant protein 1 (MCP-1) [43]. We did not analyse the concentrations of these factors in our study. However, it is possible that diabetic AT-MSCs secrete these factors, which could explain the increased formation of capillary-like structures observed in HUVECs incubated in diabetic AT-MSC-derived conditioned medium. Conducting a future study in which the VEGFR, Tie-2, CXCR1/2, IL-6R, CCR2 receptors on the surface of target endothelial cells are blocked and subsequent analysis of capillary-like tube formation could help verify their involvement in pro-angiogenic potential of HUVECs and define the crucial pro-angiogenic factors secreted by diabetic AT-MSCs (Fig. 8B).

We found that the secretome of diabetic AT-MSCs can positively stimulate pro-angiogenic events in target endothelial cells. HUVECs incubated in conditioned media derived from diabetic AT-MSC culture exhibited a higher pro-angiogenic potential compared to HUVECs incubated in conditioned media from healthy donor-derived AT-MSC culture. Furthermore, adding glucose and insulin to the culture medium of AT-MSCs derived from healthy donors subsequently had a positive effect on the pro-angiogenic potential of HUVECs exposed to the collected conditioned media. Oppositely, the other investigators reported that presence of T2D in tissue donors does not influence the capacity of MSC-secreted factors to support angiogenesis: scratches treated with conditioned media from healthy and T2D BM-MSCs both stimulated HUVEC migration over the negative control but displayed no differences in closure percentage between each other [15]. As EVs may exert pro-angiogenic effects in vitro and in vivo, we plan to conduct further experiments to compare the molecular composition and functional impact of EVs isolated from the conditioned media of healthy and diabetic AT-MSCs on target endothelial cells. This has not yet been the subject of scientific research.

In case of other therapeutic applications of MSCs, the beneficial effects observed in patients after MSC administration, can also be attributable to MSC ability to transdifferentiate into other tissue-specific cells [21] such as chondroblasts or osteoblasts. Furthermore, it has been shown that the renewal of bone tissue is centered on the interaction between angiogenic and osteogenic processes [20]. Thus, we compared the potential of AT-MSCs derived from healthy and T2D donors to differentiate into adipocytes, chondroblasts and osteoblasts (what also confirm their multipotent characteristics). During osteogenic differentiation of both populations of AT-MSCs, we did not observe any significant differences in the expression of genes related to osteogenesis or the deposition of the red-colored calcium phosphate characteristic of osteogenic differentiation between both types of AT-MSCs. Cassidy et al*.* also did not observe a significant impact of diabetes on osteogenic differentiation capacity of human BM-MSC, whereas reduced osteogenic differentiation of human diabetic AT-MSCs was reported [15, 16].

AT-MSCs derived from T2D donors exhibited lower adipogenic differentiation capacity in vitro, as indicated by the lower expression of the adipogenesis-related gene – CEBPα and a decrease in the accumulation of lipid droplets characteristic of adipogenic differentiation when compared to AT-MSCs from healthy donors. It has been demonstrated that CEBPα may promote the adipogenic differentiation program in mouse fibroblastic cells [44], what may explain observed lower adipogenic potential of diabetic AT-MSCs. Barbagallo et al*.* conducted research on human AT-MSCs from healthy and T2D donors, which were purchased from the same source as in the current study (Lonza), and demonstrated decreased expression of analyzed adipogenesis-related genes (CEBPα, FAS, FABP4, DGAT1, SCD-1, SREBP-1c), transcription factors (PPARα, PPARδ, PPARγ) and parallel lower accumulation of lipid droplets in diabetic AT-MSCs at 21 days of adipogenic stimulation when compared to healthy donor-derived AT-MSCs, which is consistent with our observations. Their results also indicated that many adipogenic gene markers were not expressed in diabetic AT-MSCs after differentiation, what may suggest that these cells do not differentiate terminally into adipocytes [24]. Decreased expression of adipogenic genes in diabetic fat cells was also presented by Dubois et al. [45], whereas Cassidy et al*.* has shown that the presence of T2D in the donor did not influence human BM-MSCs capacity to undergo adipogenic differentiation [15].

To evaluate the potential of diabetic AT-MSCs to stimulate the regeneration of injured cartilage prior to the development of ATMP, it is essential to examine their, among others, chondrogenic differentiation capacity (being one of the mechanisms of MSC pro-regenerative activity during osteoarthrosis treatment). Therefore, both populations of AT-MSCs were differentiated into chondroblasts. We observed greater expression of chondrogenesis related genes such as Sox9, ACAN 5 + 6, COL2A1 in diabetic AT-MSCs when compared to AT-MSCs isolated from healthy donors, while the histological staining indicated faster kinetics of extracellular secretion of sulphated proteoglycans characteristic of this differentiation also in diabetic cells. It has been demonstrated that a low glucose concentration (1.0 mg/ml) in the culture medium of human BM-MSCs can modulate their pro-chondrogenic potential by regulating PKC activity and TGF-β signalling [46]. Thus, the elevated level of glucose during diabetes might stimulate the pro-chondrogenic potential of AT-MSCs. However, a high concentration of glucose did not stimulate the pro-chondrogenic potential of BM-MSCs [46], as it was also observed in our study where a high glucose concentration was present in the culture/differentiation media. Wu et al*.* also confirmed positive impact of glucose on chondrogenesis—glucose at the concentration ≥ 10 mM in the culture medium induced aggrecan (proteoglycan that plays a critical role in cartilage structure and the function of joints) expression in chondrocytes through downregulation of microRNA-141-3p and parallel activation of protein kinase Cα and p38 protein in chondrocytes [47]. Interestingly, in chick MSCs high glucose concentration (30 mM) promotes chondrogenesis via PKCα and MAPK signaling pathways [48]. We are planning to conduct further mechanistic validation experiments to verify activation of chondrogenesis-related pathways such as TGF-β/Smad, Wnt/β-catenin, PI3K/Akt, and MAPK/PKCα in diabetic and healthy AT-MSCs post chondrogenic stimulation (Fig. 8C). Furthermore, the comparable expression of COL10A1, which may be involved in chondrocyte hypertrophy [49], was detected in both types of AT-MSCs. We did not observe chondrocytes hypertrophy post intraarticular administration of healthy donors-derived AT-MSCs in a pig model of cartilage-bone injury in vivo what confirm their proper chondrogenic potential [50].

In the current study, the human diabetic donors had suffered from diabetes for 10 and 20 y prior to adipose tissue collection, that may indicate adaptation of AT-MSCs to diabetes-induced oxidative stress. Variations in the demographic and clinical characteristics of individual donors, including age, BMI and comorbidities, may impact the biological potential of AT-MSCs. However, in the current study, we did not observe any significant differences between AT-MSCs in each subgroup that may indicate the “catching” of general tendencies for both types of MSCs. The other investigators observed impaired biological potential of MSCs isolated from streptozotocin-induced diabetic rats/ mice [13, 26]. In case of these animals, it is highly probable that they experienced strong oxidative stress, what may explain the observed MSC dysfunctions. Moreover, hPL, which was used to culture the MSCs, is enriched in a wide range of pro-angiogenic and pro-chondrogenic growth factors and cytokines, including EGF, FGF-2, VEGF, IGF-1, PDGF-AB/BB, BMP-2, PF4 and TGF-β1, IL-12p70, IL-17A, TNF-α and IFN-γ [51], as well as others that have not been identified. These factors may “prime” AT-MSCs and modulate their greater pro-angiogenic secretome as well as increasing the chondrogenic differentiation of diabetic AT-MSCs. Moreover, the increased blood glucose level may also promote pro-chondrogenic potential of MSCs.

To summarize, the obtained results strongly confirm that diabetic AT-MSCs (derived from donors with long-lasting diabetes) are functional cells- they represent viable, non-senescent, proliferating cells possessing morphology characteristic of MSCs. Diabetic AT-MSCs exhibited lower adipogenic differentiation potential, grater chondrogenic differentiation ability, and parallel comparable osteogenic differentiation capacity when compared to AT-MSCs derived from healthy donors. The present study provides evidence for the biological potential of AT-MSCs from T2D donors, which can be used to develop autologous ATMPs dedicated for diabetic patients. This increases the possibility of treating diabetic patients using their own cells, which is the greatest value of this study. Autologous administration of cells is safer and has a range of benefits for the patient. However, it seems crucial to include the duration of diabetes when developing donor qualification criteria. Further research is necessary to evaluate the correlation between the duration of diabetes, glycemic level, and the biological potential of AT-MSCs. It should be noted that the biological properties of two subpopulation of AT-MSCs may differ depending on the medium used to culture the cells [52].

## Conclusions

The present study provides evidence of the biological potential of diabetic AT-MSCs, which were previously considered to be dysfunctional cells. These cells can be used as an active substance in the development of autologous ATMPs. This increases the possibility of treating diabetic patients using their own cells, which is the most important achievement of our research. Preparing autologous cell products for diabetic patients is useful in avoiding the occurrence of oxidative stress that may impair biological potential of MSCs. Diabetic AT-MSCs possess greater chondrogenic and proangiogenic potential compared to AT-MSCs from healthy donors and may be use as an active substance during development of autologous ATMPs, dedicated for treatment of *e.g.* osteoarthrosis or myocardial infarction. The high proliferation rate and viability of diabetic AT-MSCs are additional arguments for the possibility of using these cells in regeneration therapies.

## Supplementary Information

Additional file 1.

## Data Availability

No datasets were generated or analysed during the current study.

## Abbreviations

ATMPs: Advance therapy medicinal products
AT-MSCs: Adipose tissue- derived mesenchymal stem/stromal cells
BM: Bone marrow
CM: Culture medium
DM: Differentiation medium
hPL: Human platelet lysate
MSCs: Mesenchymal stem/stromal cells
T2D: Type 2 diabetes
T2DM: Type 2 diabetes mellitus

## Declarations

### Use of AI-assisted technologies

The authors declare that they have not use Large Language Models during preparing of the manuscript.
