# Supplementary material for: Comparison of biological properties of human adipose tissue-derived mesenchymal stem/ stromal cells from healthy and diabetic donors: consequences for cell-based medicinal product development
Source: Cardiovasc Diabetol. 2025 Oct 29;24:413. doi: 10.1186/s12933-025-02943-x (PMC12574098; doi:10.1186/s12933-025-02943-x)
Supplement: Supplementary file 1 — Additional file 1. [file 12933_2025_2943_MOESM1_ESM.docx]

**Additional file 1**

**Comparison of biological properties of
human adipose tissue-derived mesenchymal stem/ stromal cells
 from healthy and diabetic donors:
consequences for cell-based medicinal product development**

Patrycja Dudek*, Anna Łabędź-Masłowska*, Zbigniew Madeja, Ewa Zuba-Surma

Department of Cell Biology, Faculty of Biochemistry, Biophysics and Biotechnology, Jagiellonian University, Kraków, Poland

Correspondence: Ewa Zuba-Surma, e-mail: [ewa.zuba-surma@uj.edu.pl](mailto:ewa.zuba-surma@uj.edu.pl)

* First coauthors.

**METHODS**

**Culture of AT-MSCs**

Human AT-MSCs obtained from healthy donors (PT-5006; BMI mean: 29.7; Table S1) and patients with T2D (PT-5008; BMI mean: 30.7; Table S1) were purchased from Lonza. According to the certificate of analysis provided by Lonza, AT-MSCs obtained from each donor exhibited the following multiantigenic phenotype: CD13^+^/ CD29^+^/ CD44^+^/ CD73^+^/ CD90^+^/ CD105^+^/ CD166^+^/ CD14^-^/ CD31^-^/ CD34^-^/ CD45^-^.

**Table S1.** General characteristics of adipose tissue donors. T2D – type 2 diabetes mellitus; F – female; M – male; n/a – not applicable; Negative – free of *Mycoplasma spp.* contamination; ns – no statistical significance; (*) - No information was provided by the supplier.

| Type of donors | No.  of donor  (Batch no.) | Age | Sex | BMI | Duration of diabetes | Mycoplas-ma test |
| --- | --- | --- | --- | --- | --- | --- |
| Healthy donors  (PT-5006) | 1 (0000692059) | 44 | F | 34.0 | n/a | Negative |
|  | 2 (0000672320) | 58 | M | 30.0 | n/a | Negative |
|  | 3 (0000647217) | 61 | F | 25.0 | n/a | Negative |
|  |  | Mean: **54.3** |  | Mean: **29.7** |  |  |
|  | | | | | | |
| T2D donors (PT-5008) | 1 (1F4619) | 39 | F | 33.0 | *** | Negative |
|  | 2 (1F4297) | 72 | F | 29.0 | 10 y | Negative |
|  | 3 (1F4521) | 76 | M | 30.0 | 20 y | Negative |
|  |  | Mean: **62.3** |  | Mean: **30.7** |  |  |
|  | | | | | | |
| *P  (Healthy vs. T2D donor)* | n/a | *ns* | n/a | *ns* | n/a | n/a |
|  | | | | | | |

In the current study, three types of expansion culture medium (CM) was used: i) standard CM (Control) - αMEM medium containing L-glutamine supplemented further with 5% human platelet lysate (hPL) MultiPL’100i (both from Macopharma), 2 IU / ml of heparin (Polfa Warszawa S.A.), and 1% penicillin-streptomycin solution (P/S, Thermo Fisher Scientific), ii) standard CM with the addition of 25 mM glucose (Thermo Fisher Scientific) to mimic diabetic conditions, or iii) standard CM with the addition of 25 mM glucose (ThermoFisher Scientific) and 1 µg/ml insulin (Merck) to mimic insulin-treated diabetic conditions (Table 1). The AT-MSCs were seeded in standard TC culture flasks (BD Falcon) in the CM listed above and further cultivated for 4-7 days (up to the next passage) prior to analysis including morphology, antigenic profile, viability, proliferation, oxidative stress, senescence, trilineage differentiation etc. according to the experimental scheme presented in the Fig. S1. Cells were kept under standard culture conditions (37 °C, 5% CO_2_, 95% humidity) on the HERACELL VIOS 160i CO2 incubator (Thermo Fisher Scientific). The AT-MSCs were passaged with Tryple Select Enzyme (Thermo Fisher Scientific) when the confluence of cells reached app. 80–90% and were used in the experiments from the fourth to the sixth passage. Each type of AT-MSC obtained from three individual human donors was used to replicate each experiment.

**Antigenic phenotyping of AT-MSCs**

The AT-MSCs obtained from healthy and T2D donors were passaged and subsequently resuspended in αMEM supplemented with 2% hPL (both from Macopharma), 2 IU/mL heparin (Polfa Warszawa S.A.) and further immunolabeled with monoclonal antibodies against the following selected human antigens: anti-CD19, anti-CD45, anti-CD90, anti-CD105 according to the manufacturer’s protocols for 30 min at 4°C. The used antibodies were validated with reference to an appropriate isotype control. Cells were further washed with phosphate buffered saline (PBS) without (w/o) Ca^2+^, Mg^2+^ (HyClone, Cytiva) and analysed using the LSR Fortessa flow cytometer and the FACS Diva software (Becton Dickinson). The list of used antibodies and isotype controls is presented in the Table S2.

**Table S2.** List of mouse anti-human monoclonal antibodies and isotype controls used for the antigenic phenotyping of AT-MSCs.

| Antibody against antigen/ Isotype control | Isotype | Fluorochrome | Clone | Manufacturer |
| --- | --- | --- | --- | --- |
| CD19 | Mouse IgG1, κ | FITC | HIB19 | BD Pharmingen |
| CD45 | Mouse IgG1, κ | PE | HI30 | Biolegend |
| CD90 | Mouse IgG1, κ | PE | 5E10 |  |
| CD105 | Mouse IgG1, κ | PE | 43A3 |  |
| mouse IgG1 | Mouse IgG1, κ | FITC | X40 | BD Pharmingen |
| mouse IgG1 | Mouse IgG1, κ | PE | MOPC-21 | Biolegend |

**Apoptosis and necrosis detection**

Apoptosis and necrosis evaluation was conducted using the FITC Annexin V Apoptosis Detection Kit (BD Bioscience) according to the manufacturer’s protocol. AT-MSCs after double washing with PBS w/o Ca^2+^, Mg^2+^, were resuspended in 1X binding buffer at a concentration of 10^6^ cells/ml. 100 μl of the solution transferred to a 5 ml tube was stained with FITC-conjugated Annexin V and propidium iodide (PI) and incubated for 15 min at RT in the dark. The stained cells resuspended in 1X Binding Buffer were subsequently analysed using an LSR Fortessa flow cytometer and FACS Diva software (Becton Dickinson).

**Assessment of the proliferation rate**

The AT-MSCs were seeded in a 96-well plate (Eppendorf) at the density of 10^3^ cells/well in: i) CM or ii) CM with addition of glucose (25 mM) or iii) CM with addition of glucose (25 mM) and insulin (1 µg/ml) and cultured under standard conditions (37°C, 5% CO_2_). Analysis was carried out using the Cell Counting Kit-8 (CCK-8, Merck) according to the manufacturer's protocol. Briefly, 10 μl of the CCK-8 reagent was added daily to subsequent wells containing 100 μl of culture medium and then the plate was incubated for 1 h at 37°C. The absorbance was measured at a wavelength of 450 nm using a Multiskan FC microplate photometer (Thermo Fisher Scientific) from 1 day after cell seeding up to 7 days of culture.

**Proliferation efficiency**

To compare proliferation efficiency of cells (rated as percentage of culture well coverage), AT-MSCs obtained from healthy and T2D donors were seeded in a 6-well plates (Eppendorf) with a density of 10, 100, 200, 400 or 1000 cells per well in: i) CM, or ii) CM with addition of glucose (25 mM), or iii) CM with addition of glucose (25 mM) and insulin (1 µg/ml). Cells were cultured under standard conditions (37°C, 5% CO_2_). The medium was replaced every 3 to 4 days. After 21 days, cells were fixed with 4% paraformaldehyde (CHEMPUR) and stained with 0.4% trypan blue solution (HyClone). After staining, percentage of cell coverage of the culture wells was calculated using Fiji ImageJ (Schneider et al., 2012).

**Assessment of senescence**

Both types of AT-MSCs were seeded in a 6-well plate (Eppendorf) at the density of 4.0 × 10^3^ cells/cm^2^ in: i) CM, or ii) CM with addition of glucose (25 mM), or iii) CM with addition of glucose (25 mM) and insulin (1 µg/ml) and cultured under standard conditions (37°C, 5% CO2). AT-MSC senescence was analysed at 4 days after cell seeding using the Senescence β-Galactosidase Staining Kit (Cell Signaling Technology) according to the manufacturer's protocol. Briefly, cells were incubated with a staining mixture overnight. The blue-stained (β-galactosidase-positive; characteristic for senescent cells) and unstained cells (nonsenescent cells) were visualised using an Olympus IX81 microscope equipped with a MicroPublisher 3.3 RTV camera (Olympus).

**Capillary-like tube formation assay**

AT-MSCs (at 3 days post seeding; confluence reached app. 80%) were washed with PBS w/o Ca^2+^, Mg^2+^ (HyClone) and incubated in αMEM medium with L-glutamine (Macopharma) supplemented with 1% human serum albumin (Grifols) and 1% P/S solution (ThermoFisher Scientific) for 24 h. The conditioned media were collected and stored at -80°C. On the day of analysis, the conditioned media were thawed at 37°C until only a small clump of ice remained and centrifuged at 350xg, for 10 min at 4°C to remove cells. The supernatants were transferred into new 50 ml tubes (Sarstedt) and centrifuged at 500xg for 20 min at 4 ° C to remove the remaining cells and cell debris and subsequently centrifuged at 2,000xg for 20 min at 4°C to remove smaller cellular debris and apoptotic bodies.

To perform a Matrigel-based tube formation assay, a 24-well plate (Eppendorf) was coated with Matrigel matrix growth factor reduced (BD Pharmingen; 100 μL/well) and incubated at 37°C for 30 min. Before the experiment, HUVECs (Lonza) were cultured in EGM-2MV Microvascular Endothelial Cell Growth Medium-2 BulletKit (Lonza). To evaluate the pro-angiogenic properties of the AT-MSC secretome, the HUVECs were seeded at a density of 7.5 × 10^4^ cells/ well in the collected conditioned media (derived from AT-MSC culture). Tube formation was investigated with a Leica DMI6000B microscope (ver. AF7000) and analysed with ImageJ software.

**Detection of the marker of oxidative stress- 8-hydroxy-2’-deoxyguanosine (8OHdG)**

From both types of AT-MSC cultured in three types of CM (as described in Table 1), total DNA was isolated using the GeneMATRIX Cell Culture DNA Purification Kit (EURx) according to the manufacturer's protocol. AT-MSCs obtained from healthy and T2D donors cultured in standard CM supplemented with 10 ng/ml IL-1β (BioLegend) under hypoxia conditions (37°C, 5% CO_2_, 5% O_2_) for 48 h was used as a positive control. Nano Drop OneC (Thermo Fisher Scientific) was used to measure DNA concentration.

The concentration of the marker of oxidative stress- 8OHdG in both types of cells was determined using a commercially available DNA Damage Competitive ELISA Kit (Thermo Fisher Scientific) according to the manufacturer’s protocol. The inter and intra-assay coefficients of variability for the analysis were 9.9% and 9.0%, respectively. Absorbance values were measured at 450 nm using the Multiscan FC plate reader (ThermoFisher Scientific). The standard curve of the relationship between the values of 1/OD450 (x axis) and the concentration of 8OHdG (y axis) was created. Subsequently, the concentration of 8OHdG in the analysed samples was calculated from the linear equation (for which R^2^ was around 0.95).

**Adipogenic, osteogenic and chondrogenic differentiation of AT-MSCs**

In the case of adipogenic and osteogenic differentiation, 1.9 × 10^4^ cells/ well were seeded in a 12-well plate (BD Falcon) in the standard CM or CM with addition of glucose (25 mM), or CM with addition of glucose (25 mM) and insulin (1 µg/ml) as described in the
Table 1. To induce chondrogenic differentiation, micro mass cultures were generated by seeding 5 µl droplets of cell solution (one droplet contained 4 × 10^4^ cells) in a 12-well plate (BD Falcon) and incubated for 30 min under high humidity conditions. Subsequently, cells were flooded with the standard CM or CM with addition of glucose (25mM), or CM with addition of glucose (25 mM) and insulin (1 µg/ml) (Table 1). After 24 h of AT-MSC cultivation, culture medium was replaced with: StemPro Adipogenesis Differentiation Kit, StemPro Osteogenesis Differentiation Kit, or StemPro Chondrogenesis Differentiation Kit (Thermo Fisher Scientific), which were used in three variants: i) without additional ingredients (control DM), or with the addition of ii) 25 mM glucose (Thermo Fisher Scientific), or iii) 25 mM glucose (Thermo Fisher Scientific) and 1 µg/ml insulin (Merck) as described in Table 1 (manuscript). Cultures were re-fed every 3 to 4 days. At 3, 7, 14, and 21 days, adipogenic, osteogenic, and chondrogenic differentiation was confirmed by i) mRNA analysis, and ii) histological staining.

**Gene expression analysis**

Total RNA was isolated using the Universal RNA/ miRNA Purification Kit (EURx) according to the manufacturer's protocol. RNA concentration and purity were assessed by Nano Drop OneC (Thermo Fisher Scientific). The isolated RNA was reverse transcribed into cDNA using NG dART RT Kit (EURx) according to the manufacturer's protocol and using C1000 Touch ThermalCycler (Bio-Rad). Conditions of the RT reaction: 25 °C for 10 min, 50 °C for 50 min, 85 °C for 5 min. Subsequently, the expression of genes related to adipogenesis (CEBPα, PPRγ), osteogenesis (osteocalcin, osteopontin, Runx2) and chondrogenesis (SOX9, ACAN, COL2A1, COL10A1, HPLN) was examined by real-time PCR using QuantStudio 6 Flex Real-Time PCR System (Thermo Fisher Scientific). The sequences of the primers are presented in Table S3. β2-microglobulin was used as a control housekeeping gene. The relative level of gene expression was determined using the comparative ΔΔC_T_ method. To evaluate statistically significant differences between AT-MSCs obtained from healthy and T2D donors in the used media (described in Table 1), the level of gene expression after differentiation was compared to the level of gene expression before differentiation (day 0) for both types of cells independently.

**Table S3**. Oligonucleotide sequences used for RT-PCR.

| Gene | Sequences |
| --- | --- |
| β2-microglobulin | (F) 5’ AAGGACTGGTCTTTCTATCTC 3’ |
|  | (R) 5’ GATCCCACTTAACTATCTTGG 3’ |
| *CEBPα* | (F) 5’ AGGTTTCCTGCCTCCTTCC 3′ |
|  | (R) 5’ CCCAAGTCCCTATGTTTCCA 3′ |
| *PPRγ* | (F) 5’ AGGCGAGGGCGATCTTGACAG 3′ |
|  | (R) 5’ GATGCGGATGGCCACCTCTTT 3′ |
| *Osteocalcin* | (F) 5’ CGCCTGGGTCTCTTCACTAC 3′ |
|  | (R) 5’ CTCACACTCCTCGCCCTATT 3′ |
| *Osteopontin* | (F) 5’ ACTCGAACGACTCTGATGATGT 3′ |
|  | (R) 5′ GTCAGGTCTGCGAAACTTCTTA 3′ |
| *Runx2* | (F) 5’ GGAGTGGACGAGGCAAGAGTTT 3′ |
|  | (R) 5’ AGCTTCTGTCTGTGCCTTCTGG 3′ |
| *SOX9* | (F) 5’ CTCTGGAGACTTCTGAACG 3’ |
|  | (R) 5’ AGATGTGCGTCTGCTC 3’ |
| *ACAN* | (F) 5’ CTACACGCTACACCCTCGAC 3’ |
|  | (R) 5’ ACGTCCTCACACCAGGAAAC 3’ |
| COL2A1 | (F) 5’ GAAGAGTGGAGACTACTGG 3’ |
|  | (R) 5’CAGATGTGTTTCTTCTCCTTG 3’ |
| *COL10A1* | (F) 5’ GCTAGTATCCTTGAACTTGG 3’ |
|  | (R) 5’ CCTTTACTCTTTATGGTGTAGG 3’ |
| *HPLN* | (F) 5’ ATGGCCGTTTTTACTATCTG 3’ |
|  | (R) 5’ CAATCTGAGCACCATCATTG 3’ |

**Histological staining**

Cells were washed with PBS (HyClone, Cytiva) and fixed with 4% paraformaldehyde (CHEMPUR) for 30 min at RT. To demonstrate the presence of lipid droplets characteristic for adipogenic differentiation, fixed cells were rinsed twice with distilled water, and then incubated with 60% isopropanol (POCH) for 15 min. After incubation, isopropanol was removed and cells were immediately stained with 1% Oil Red O solution (Merck) for 15 min. In the case of osteogenic differentiation, the fixed cells were rinsed twice with distilled water and stained with 2% Alizarin Red S solution (Merck) to indicate calcium phosphate deposition. To evaluate the presence of chondroitin sulphate in differentiated chondrogenic culture, fixed cells were rinsed with PBS and stained with Alcian Blue Staining Solution (Merck) for 30 min. Subsequently, the cells were washed three times with 0.1 N HCL (CHEMPUR). Then, to neutralise the acidity, distilled water was added. After histological staining, cells were visualised using an Olympus IX81 microscope equipped with a MicroPublisher 3.3 RTV camera (Olympus).

**Statistical analysis**

Statistical analysis was performed using GraphPad Prism 8.4 software (Graph Pad Software). Data are presented as the mean ± SD or means (on heat maps). Each experiment was conducted in triplicate using three independent replicates. One unit include one cell sample derived from one type of AT-MSCs cultured in one medium. For experiments with Gaussian distribution results, two-way ANOVA followed by NIR-Fisher or Tukey’s multiple comparisons was used to determine the statistical significance. For non-parametric probes, the Kruskal Willis test with Uncorrected Dunn’s test as a post hoc test was used. For supplementary data, an unpaired t-test was used. Results were considered statistically significant when *p< 0.05; **p<0.01; ***p<0.001. Biostatistical consultation was performed to assess the number of biological replicates per group.

**ADDITIONAL FIGURES**

**Figure S1**

**
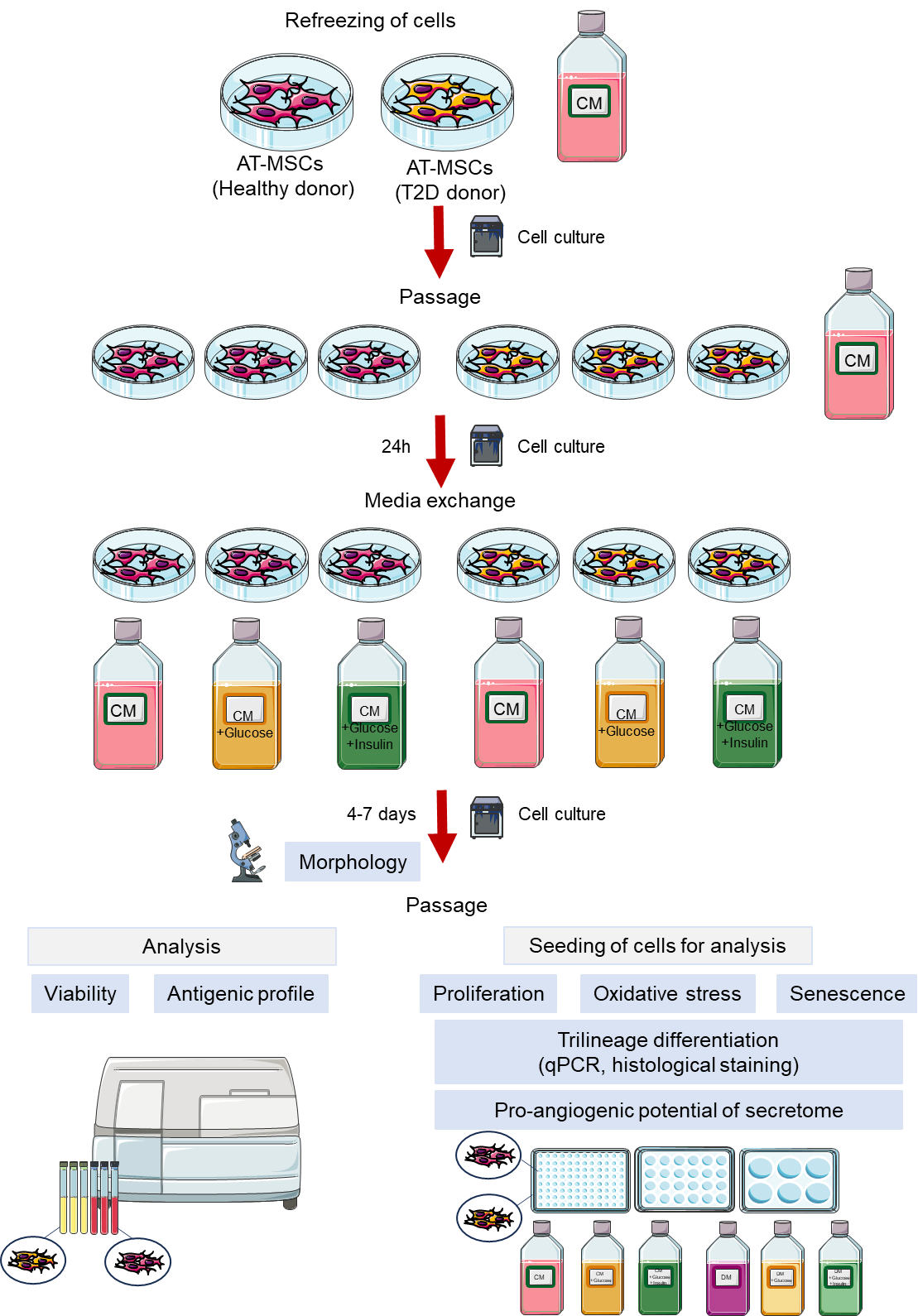
**

**Fig. S1**. Experimental layout of the study. CM - culture medium; DM - differentiation medium.

**Figure S2**

**
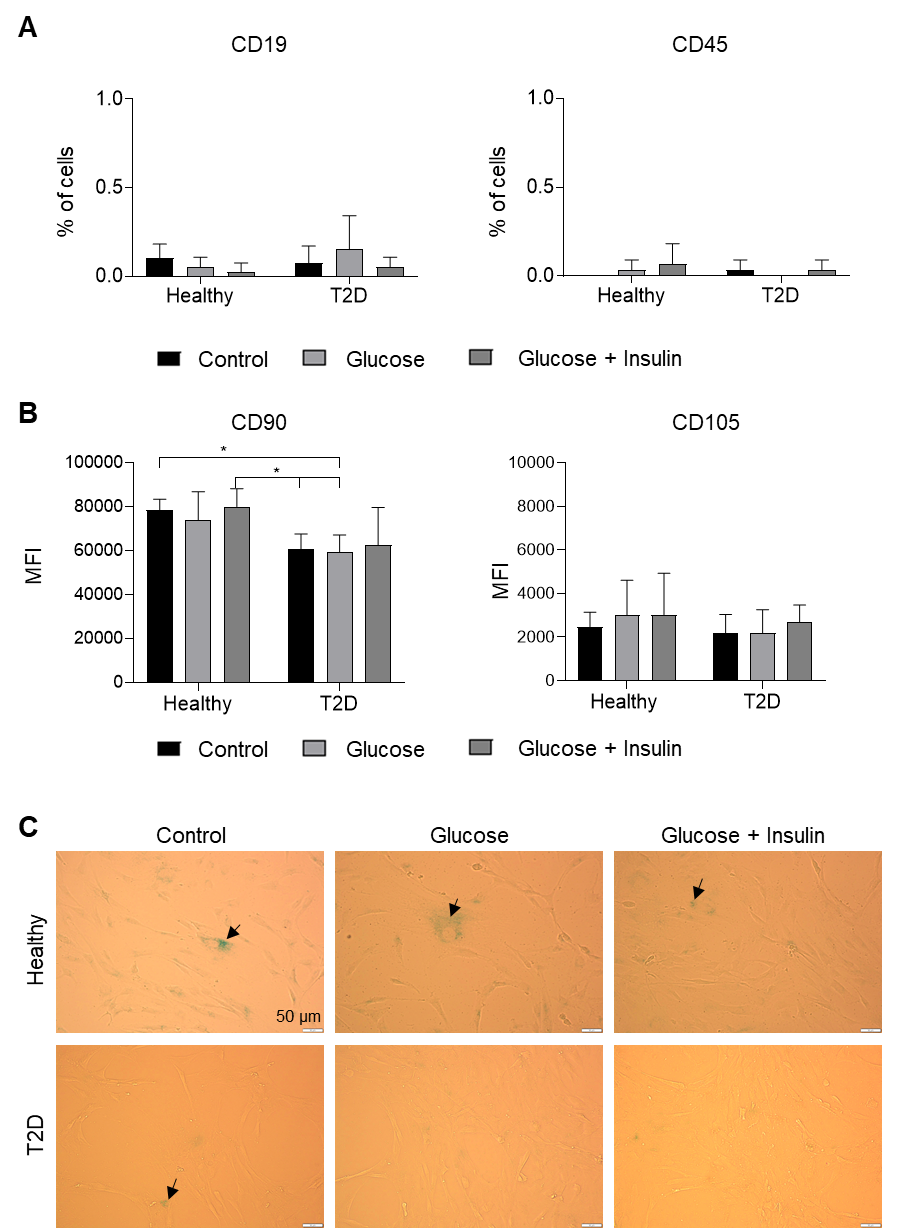
**

**Fig. S2.** Biological characteristics of AT-MSCs derived from healthy and T2D donors.
**A** Quantitative data representing the percentage content of positive AT-MSCs for CD19 and CD45. Results are presented as mean ± SD, n=3 (biological replicates). **B** Mean fluorescence intensity (MFI) of AT-MSCs stained against the CD90 and CD105 markers by flow cytometry. Results are presented as mean ± SD, n=3 two-way Anova with NIR Fisher test as post hoc.
*p< 0.05. **C** Representative images of both types of AT-MSCs at 4 days after seeding stained with Senescence β-Galactosidase Staining Kit. Blue-stained cells corresponded to senescent cells (β-galactosidase-positive cells), while unstained cells are non-senescent (β-galactosidase-negative cells). Scale bars: 50 µm. Healthy – healthy donor; T2D – diabetic donor.

**Figure S3**

**
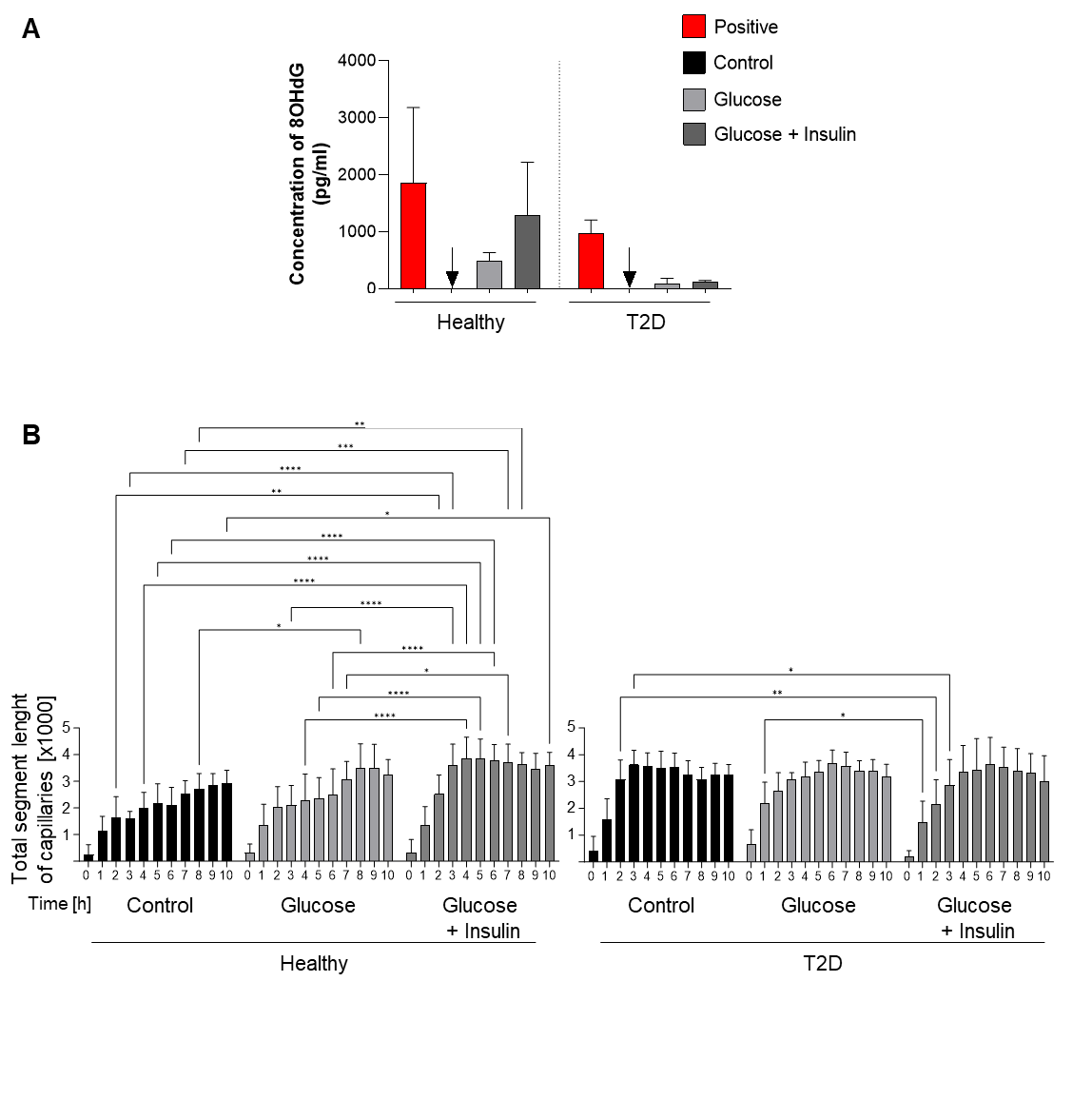
**

**Fig. S3.** Biological characteristics of AT-MSCs derived from healthy and T2D donors.
**A** Evaluation of the concentration of the 8OHdG marker of oxidative stress by competitive ELISA. Higher 8OHdG values correlate with higher oxidative stress in cells. Data are presented as mean ± SD, n= 2 (biological replicates); One-way Anova with the NIR Fisher test as post hoc (*p< 0.05; **p<0.01) was used. Positive – positive control. **B** Quantitative analysis of the pro-angiogenic potential of the AT-MSC secretome presented as total segment length of capillaries (created by HUVECs) per microscopic field at different time points, from the start of the assay (0 h) until 10 h. Results are presented as mean ± SD, n= 3 (biological replicates); two-way with the NIR Fisher test as post hoc. Healthy – healthy donor; T2D – diabetic donor.

**Figure S4**

**
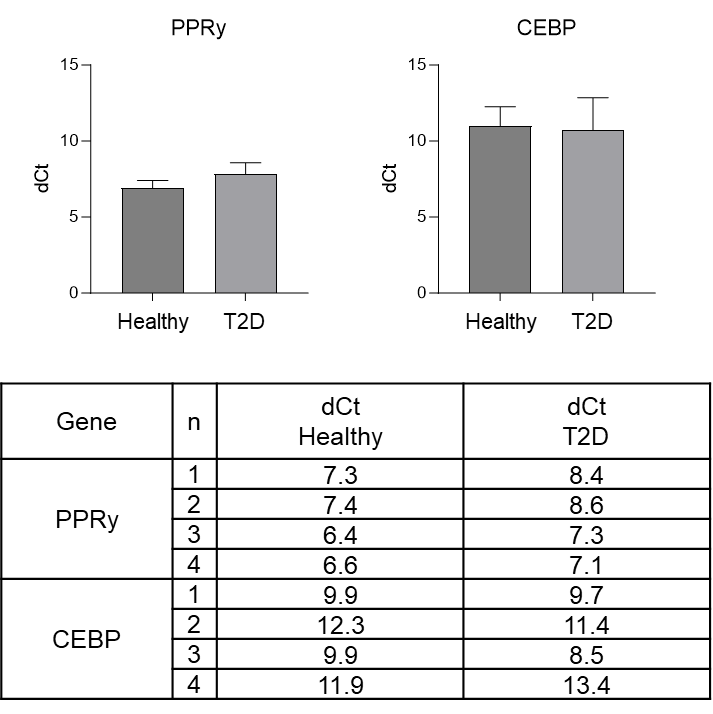
**

**Fig. S4.** Expression (dCt) of genes related to adipogenesis (PPR-γ and CEBPα) in AT-MSCs obtained from healthy and T2D donors directly before differentiation. Results are presented as mean ± SD, n= 4 (including 3 biological replicates); unpaired t-test. dCt values for individual biological replicates for the analyzed genes are presented in the table. Healthy – healthy donor; T2D – diabetic donor.

**Figure S5**

**
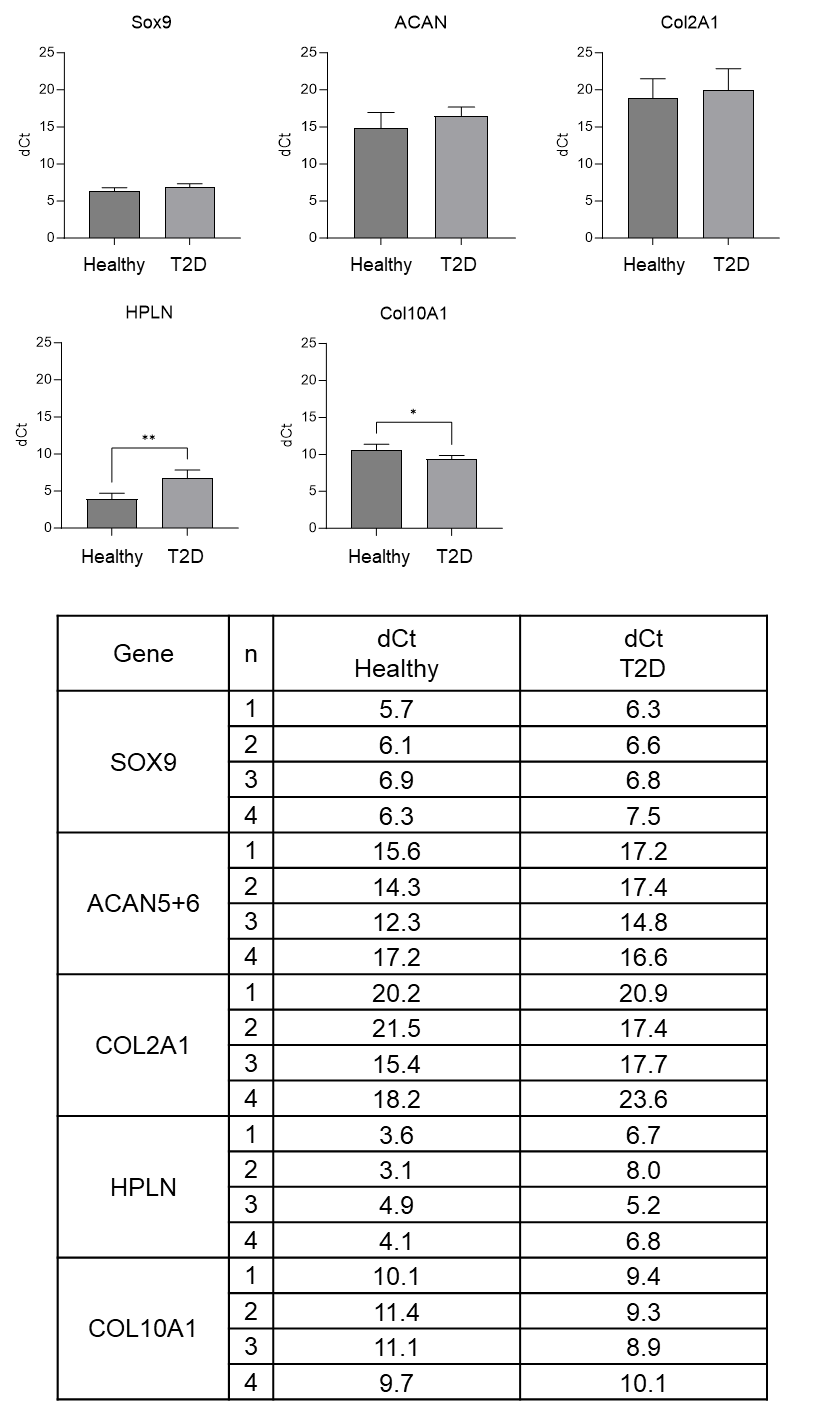
**

**Fig. S5.** Expression (dCt) of genes related to chondrogenesis (SOX9, ACAN5+6, COL2A1, HPLN and COL10A1) in AT-MSCs obtained from healthy and T2D donors directly before differentiation. The results are presented as mean ± SD, n= 4 (including 3 biological replicates); unpaired t-test. dCt values for individual biological replicates for the analyzed genes are presented in the table. *p< 0.05; **p<0.01. Healthy – healthy donor; T2D – diabetic donor.

**Figure S6**


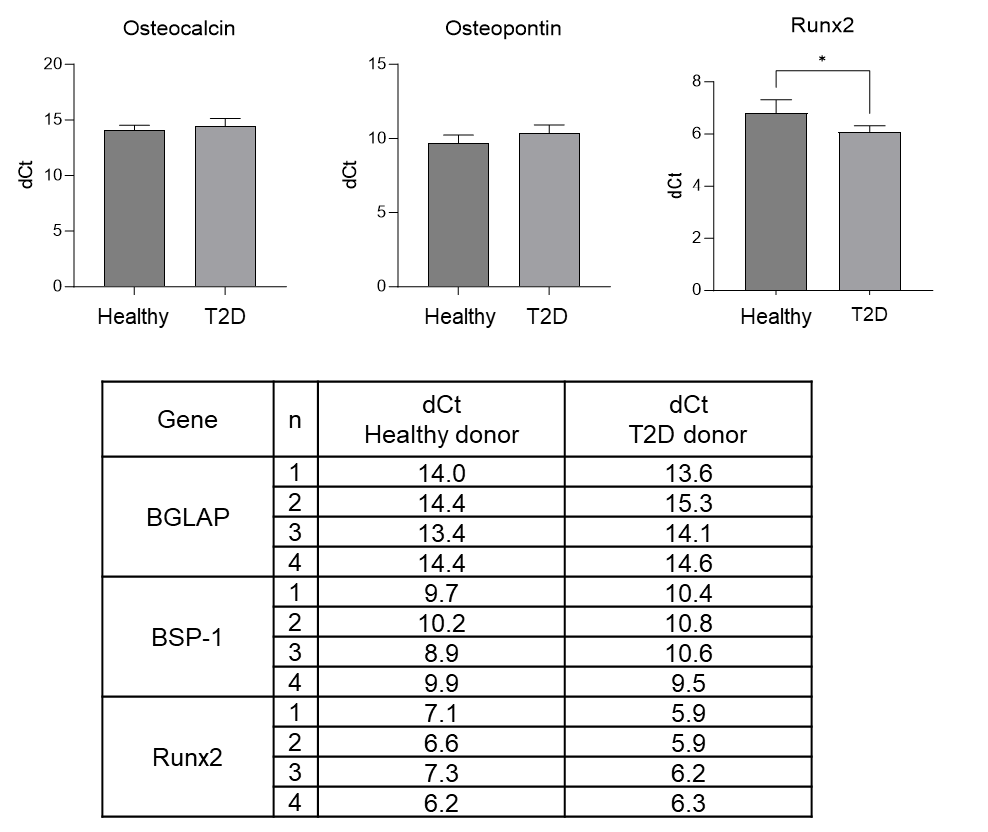


**Fig. S6.** Expression (dCt) of genes related to osteogenesis (osteocalcin, osteopontin and Runx2) in AT-MSCs obtained from healthy and T2D donors directly before differentiation. The results are presented as means ± SD, n= 4 (including 3 biological replicates); unpaired t-test. dCt values for individual biological replicates for the analyzed genes are presented in the table. *p< 0.05. BGLAP, osteocalcin; BSP-1, osteopontin. Healthy – healthy donor; T2D – diabetic donor.

**Figure S7**


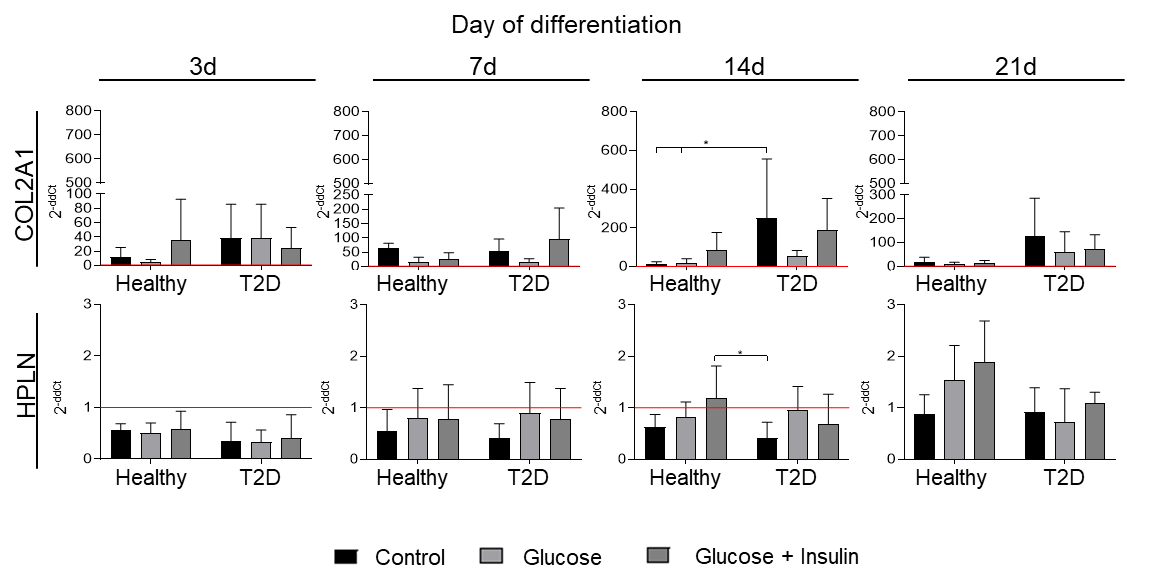


**Fig. S7.** Chondrogenic differentiation of AT-MSCs obtained from healthy and T2D donors. The AT-MSCs were cultured in standard chondrogenesis stimulating medium StemPro Chondrogenesis Differentiation Kit (Chondro-DM, Control), Chondro-DM with the addition of 25 mM glucose (Glucose), or Chondro-DM with the addition of 25 mM glucose and 1 µg/ml insulin (Glucose + Insulin) for 3, 7, 14 and 21 days. The graphs showed the expression of genes related to chondrogenesis (COL2A1 and HPLN). The fold differences in the expression (2^-ddCt^) of the analyzed genes in undifferentiated cells (on day 0) separately for AT-MSCs derived from healthy donors and T2D were calculated as 1.0 and marked by a solid red line. Results are presented as mean ± SD, n= 3 (biological replicates); two-way with the NIR Fisher test as post hoc for HPLN; Kruskal Willis test with the uncorrected Dunn test as post hoc test for COL2A1, were used for statistical analysis. *p< 0.05; **p<0.01; ***p<0.001. COL2A1, collagen type II alpha 1 chain; COL10A1, collagen type X alpha 1 chain; HPLN, proteoglycan link protein. Healthy – healthy donor; T2D – diabetic donor.

**References**

1. Schneider CA, Rasband WS, Eliceiri KW. NIH Image to ImageJ: 25 years of image analysis. Nature Methods. 2012; 9: 671–5.
